# An Electrospray Sequential Mass Spectrometry Fragmentation Scheme of Erythromycin A and Its Application for the Elucidation of the Structures of Its Natural Co-Metabolites

**DOI:** 10.3390/molecules31060928

**Published:** 2026-03-11

**Authors:** Candy Jiang, Paul J. Gates

**Affiliations:** School of Chemistry, University of Bristol, Cantock’s Close, Bristol BS8 1TS, UK; candy.jiang@bristol.ac.uk

**Keywords:** macrolide, polyketide antibiotics, erythromycin, structural elucidation, sequential mass spectrometry, fragmentation scheme, new erythromycin

## Abstract

Natural products such as polyketides are a fertile target for drug discovery. Methodologies relating to discovery, metabolism, synthesis and biosynthesis of polyketides have evolved considerably since they were first studied in the early 20th century. The antibiotic erythromycin, produced by the *Streptomyces erythreus* bacteria, was the first of the macrolide natural products to be discovered in 1952. The biosynthesis of erythromycin is catalysed by a large multifunctional enzyme, which constructs the polyketide intermediate that is acted upon by tailoring enzymes to produce the final construct. It is during this process that molecular diversity is generated, and commercial samples of erythromycin tend to be mixtures of co-metabolites. To fully identify these compounds, a full fragmentation scheme of the main component (erythromycin A) is required, which is absent from the literature. In this study, accurate-mass sequential mass spectrometry is used to propose a fragmentation scheme which is then used to assign structures to eight co-metabolites including the identification of a previously unpublished form of erythromycin. This clearly demonstrates the successful application of the methodology.

## 1. Introduction

**Figure 1:**
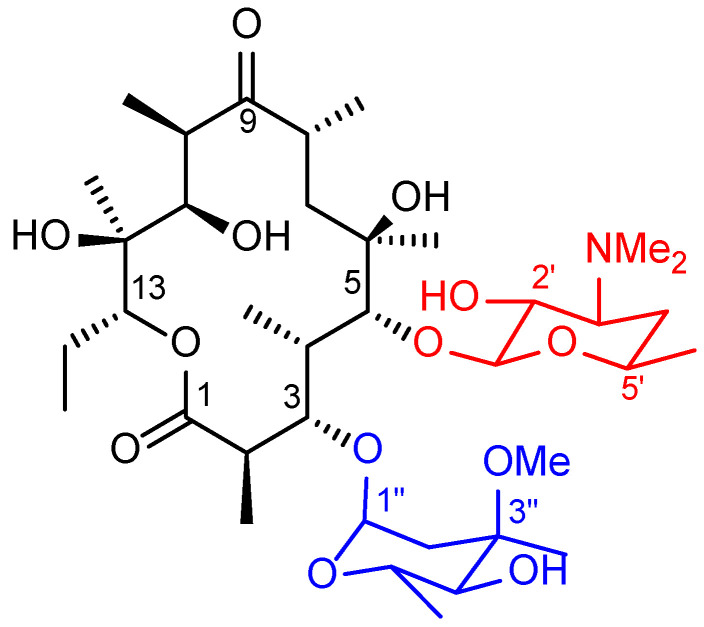
The chemical structure of the 14-memberred macrolide, erythromycin A, with the carbon skeleton numbered. Substituent sugar moieties are D-desosamine (red) and L-cladinose (blue).

Natural products are highly sought after for their wide-ranging pharmacological activities [1,2]. There is an increased need to find new chemical entities for drug discovery, especially with the prevalence of issues relating to antibiotic resistance. Polyketides are one of the major classes of natural products which can be distinguished from other natural products by the alternating carbonyl and methylene groups along their carbon skeleton. Figure 1 shows the structure of the polyketide macrolide antibiotic erythromycin A [3,4]. They are secondary metabolites, the vast majority of which are produced by bacteria and fungi species, as well as some plants, molluscs and algae [4,5]. Their biosynthesis involves the catalysis of acyl-Coenzyme A precursors by polyketide synthase (PKS) enzymes, in a process analogous to fatty acid biosynthesis. The polyketide compound class demonstrates high structural and functional diversity with over 10,000 fully characterised compounds distributed across of variety of subclasses [6], such as ansamycins, macrolides, polyenes and enediynes [7].

Some of the most prescribed polyketides are from the macrolide subclass which contains a macrocyclic lactone ring connected to deoxy sugars via glycosidic bonds [8]. The lactone ring is typically 14-, 15- or 16-membered, which means the macrolides can be classified based on the number of atoms in the lactone ring [9]. These natural macrolides are biosynthesised and isolated from *Micromonospora* and *Streptomyces* bacterial species [8]. Erythromycin A (EryA, Figure 1) is an example of a macrolide antibiotic produced by the *Streptomyces erythreus* bacteria. It was first isolated from a soil sample on the island of Panay located in the Philippines [10]. EyA is one of the most prescribed antibiotics for ailments such as chest infections, eye infections, various skin conditions, and sexually transmitted infections. It is currently on the World Health Organization’s list of essential medicines [11]. Commercial samples of erythromycin contain a mixture of up to six structurally similar compounds or analogues (see Supplementary Information Figure S1 and Table S1 for more details).

The efficacy of EryA is limited by its lack of stability under acidic conditions [12,13]. When exposed to acid, EryA decomposes through an intramolecular dehydration, producing two internally cyclised byproducts (see Figure S2) [14,15,16] with further decomposition resulting in hydrolysis of the cladinose sugar moiety leading to gastrointestinal problems [17]. The products of this decomposition express reduced biological activity [14]. Decomposition within the stomach due to gastric acid is avoided either due to pharmaceutical-grade EryA being prescribed in an acid-resistant coating, for example, using an enteric capsule in a delayed release formulation [18] or by post-biosynthetic editing to create an EryA pro-drug (e.g., erythromycin stearate) to ensure it passes through the stomach intact [19].

The current literature for MS studies of EryA tends to concentrate on detection in environment and food samples using existing knowledge of fragmentation. For example, using LC-MS/MS for studies of residues in salmon [20,21], trout [22], honey [23], chicken and eggs [24]. There are also studies on degradation products and derivatives of EryA [25,26,27,28,29]. In all cases, no complete fragmentation pathway is presented or referred to. Previous studies in 1999, using collision-induced dissociation (CID) sequential mass spectrometry (MS^n^), successfully elucidated the losses of the appropriate hydroxyls, sugars, and some initial ring fragmentations [30,31]. Mechanisms for the losses of the sugar moieties (desosamine and cladinose) were proposed and one of the key discoveries made by this study was the determination of the position for the first loss of water being from the C-9 position of the lactone ring, as opposed to being a direct hydroxyl elimination from the C-6, C-11 or C-12 positions. In this mechanism, the C-6 hydroxyl acts as a nucleophile, attacking the carbonyl to form an internal ring. A separate study in 2002 presented a full MS^n^ fragmentation scheme of a biosynthetic precursor of EryA, erythronlide B [32].

The first ring opening fragment was found to correspond to a loss of 58 Da (matching to C_3_H_6_O) which shifted to a loss of 44 Da (matching to C_2_H_4_O) for the analysis of analogue Ery1 (see Figure S3) and to a loss of 86 Da (matching to C_5_H_10_O) for Ery2. This corresponds to the replacement of the ethyl group at C-13 with a methyl group [30]. Therefore, it was concluded that the first ring-opening fragment was due to the loss of the starter acid residue used by the PKS for biosynthesis. This had immediate implications in structural elucidation of EryA analogues as it allowed for the rapid and unequivocal identification of the starter acid. Changing the starter acid is one of the methods used to create novel EryA derivatives [33,34,35]. Despite the success of these studies, there remains a limited understanding of the fragmentation pathway of EryA in the literature.

Efforts to elucidate other ring fragments have not been successful to date, and as a result the full fragmentation pathway has not been published in the literature. This study presents a much more complete fragmentation pathway for EryA than that in the existing literature, which is then used to identify several co-metabolites present in a commercial (non-pharmaceutical) sample. This analysis helps us to better understand how small changes to the structure of polyketides can predictably change the fragmentation, in turn revealing more details about the fragmentation pathways of EryA. If the structure of an unknown erythromycin derivative can be elucidated by comparison with the EryA MS^n^ spectra, then this would suggest that MS^n^ has potential as a technique for characterising the products of metabolic studies, combinatorial biosynthesis, PKS engineering, and even semi-synthetic approaches to new erythromycin analogues.

## 2. Results and Discussion

The commercial sample of EryA analysed in this study was not 100% pure. The direct infusion full-scan spectrum is shown in the Supplementary Materials (Figure S4) and here it is clear that the spectrum is dominated by the ion at *m*/*z* 734 which is the protonated ion for EryA [M + H]^+^ measured to within 1 ppm mass accuracy. This is the precursor ion (PI) for the initial MS^n^ study. There are several other peaks present in the spectrum at *m*/*z* 748, 720 and 718. These are listed in Table S2 with their proposed formulae, and the analysis of these components will be the focus of the later sections of this study.

### 2.1. Elucidation of the Fragmentation Pathways of Erythromycin A

**Figure 2:**
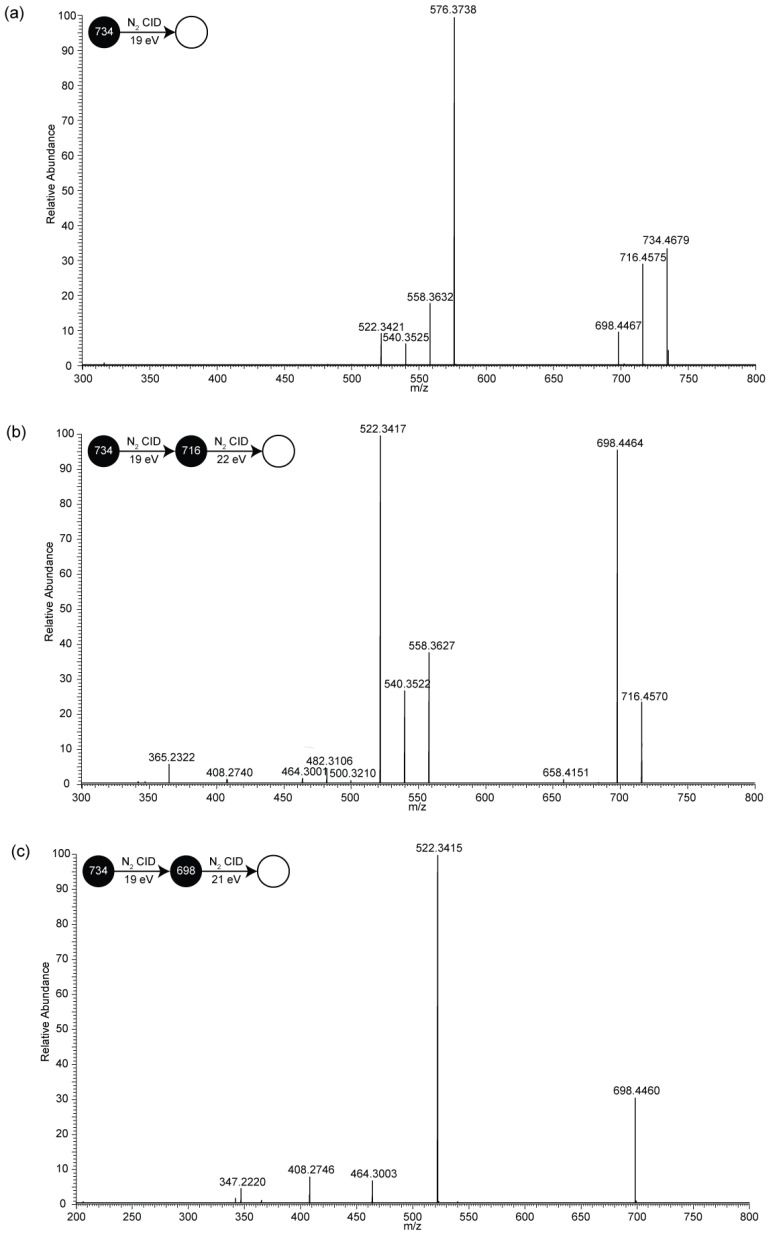
ESI-CID-MS^n^ spectra of EryA (Mw 733). Spectrum (**a**) is the MS/MS of the [M + H]^+^ (PI = *m*/*z* 734), spectra (**b**) are MS^3^ of *m*/*z* 716 and (**c**) is the MS^3^ of *m*/*z* 698. The collision energies and MS^n^ sequences are displayed on the spectra.

**Scheme 1:**
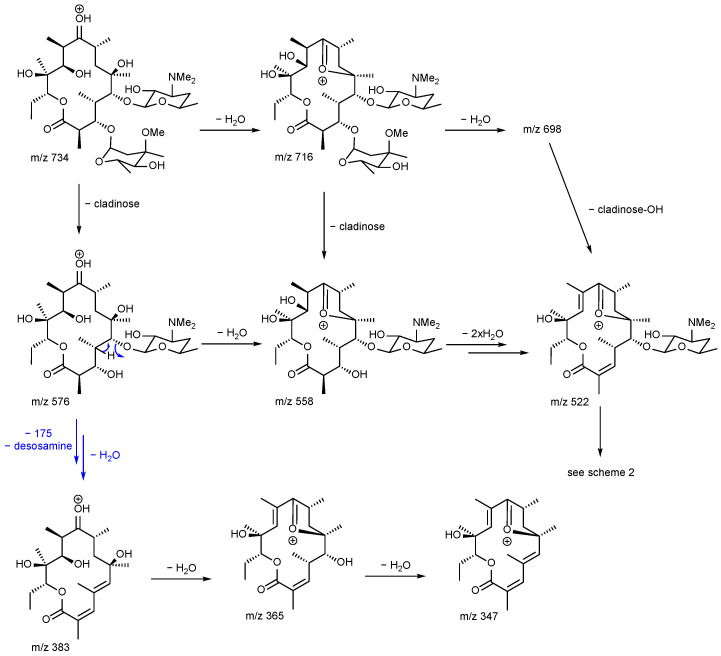
The proposed fragmentation route for EryA (PI *m*/*z* 734) showing the initial losses of water and the cladinose sugar moiety to generate product ion *m*/*z* 522. The further fragmentation of *m*/*z* 522 is detailed in Scheme 2.

Figure 2a shows the MS/MS spectrum of the [M + H]^+^ of EryA (PI *m*/*z* 734) with six product ions formed. Figure 2b,c show two MS^3^ analyses of the two product ions with the highest masses from the MS/MS analysis. Accurate masses for all product ions are contained in Table S3. The first two product ions observed are (*m*/*z* 716 and 698) due to successive losses of H_2_O from the precursor ion. The first neutral loss will presumably follow the same mechanism as previously published, resulting in internal cyclisation and the removal of the C-9 carbonyl [31]. Determining the position of the hydroxyl group associated with the second water loss is difficult as there are two remaining hydroxyls on the lactone ring. The third product ion (*m*/*z* 576) corresponds to the loss of the cladinose sugar from C-3, which is in competition with the first two water losses. The MS^3^ analysis of *m*/*z* 576 (see Figure S5a) is dominated by three consecutive water losses to generate product ions at *m*/*z* 558, 540 and 522, the third of which is only possible due to the availability of the newly formed hydroxyl at the C-3 position, which replaces the cladinose sugar. This is followed by losses of propionate from these ions to generate product ions *m*/*z* 500, 482 and 464. There is also a product ion at *m*/*z* 383 due to losses of both sugar moieties and water (see Scheme 1). The MS^3^ spectra show several additional lower mass product ions which will be discussed in more detail below.

The MS^3^ analysis of *m*/*z* 716, shown in Figure 2b, reveals that the loss of cladinose can also occur in between the first and second water losses to generate product ion *m*/*z* 558 (see Scheme 1), which is then followed by two additional water losses (*m*/*z* 540 and 522). The product ions at *m*/*z* 500, 482 and 464 are due to loss of the 58 Da propionyl starter acid unit from *m*/*z* 558, 540 and 522 respectively [31]. This loss was confirmed by MS^3^ and MS^4^ analysis. The odd-massed product ion at *m*/*z* 365 contains no nitrogen. Losses of the three waters and both sugars result in a product ion with this mass (see Scheme 1), which in turn loses another water to generate the product ion at *m*/*z* 347. Interestingly, the loss of the propionyl starter acid unit only occurs after the removal of the cladinose sugar and at least one H_2_O. It has previously been theorised that migration of the C-12 hydroxyl to C-9 would enable the loss of the propionyl starter acid by formation of a carbocation at C-11 [36].

**Scheme 2:**
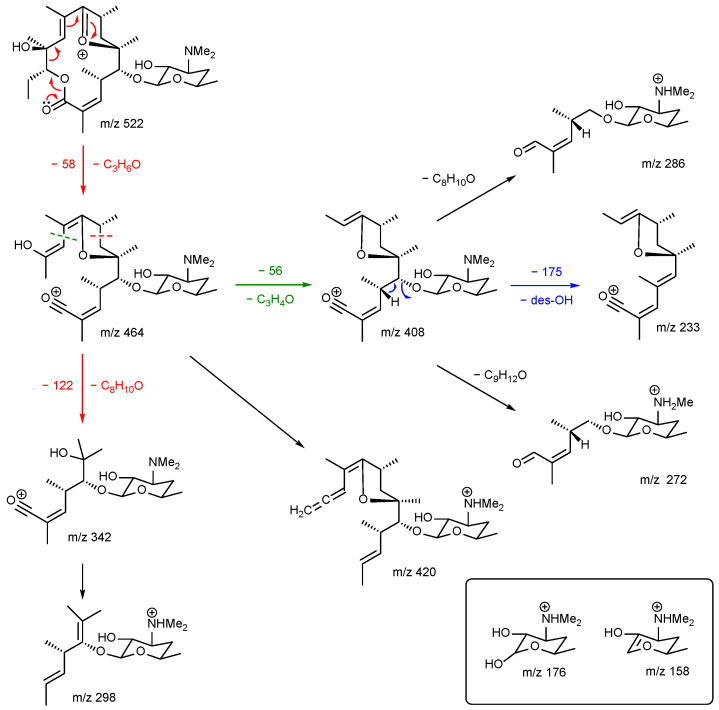
The sub fragmentation of *m*/*z* 522 generated through the MS^n^ analysis (see Figure 3). The structures in the box show the two protonated desosamine ions. The structures of product ions *m*/*z* 286 and 272 are proposals based purely on the accurate-mass formulae determination.

**Figure 3:**
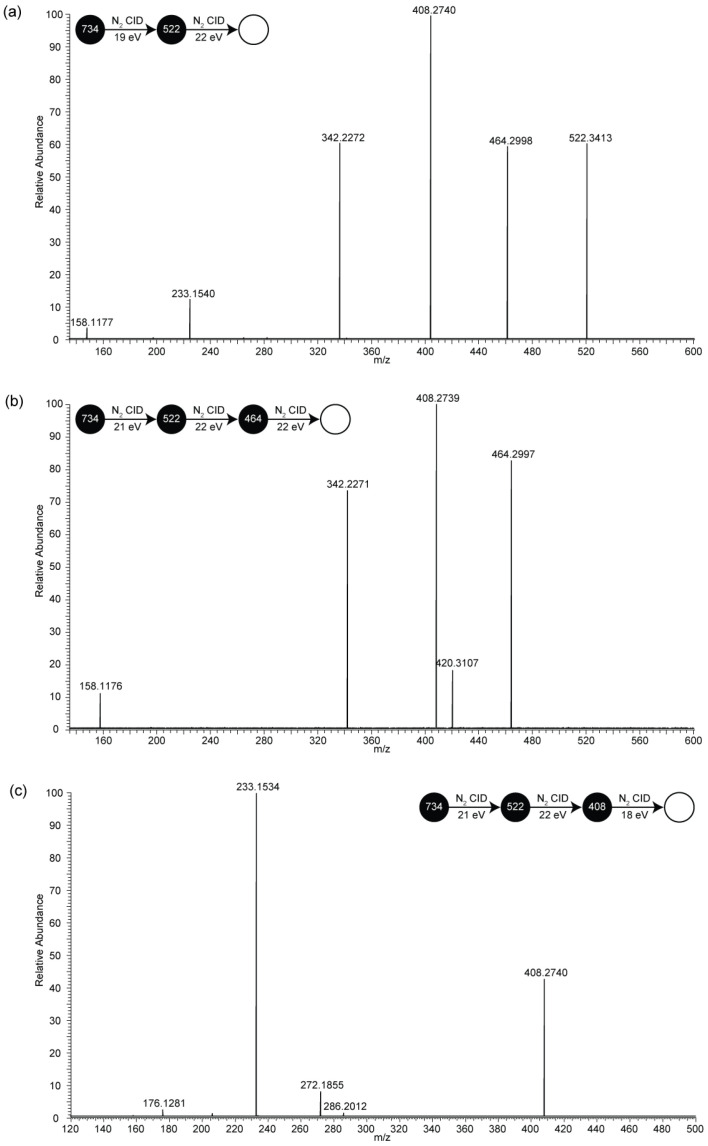
ESI-CID-MS^n^ spectra of EryA. Spectrum (**a**) is the MS^3^ of *m*/*z* 522, spectrum (**b**) is the MS^4^ of *m*/*z* 464 and spectrum (**c**) is the MS^4^ of *m*/*z* 408. The collision energies and MS^n^ sequences are displayed on the spectra.

The MS^3^ analysis of *m*/*z* 522, see Figure 3a, shows five product ions. The first is *m*/*z* 464 which is due to the loss of the propionate starter acid [30,31]. This ion then generates product ion *m*/*z* 342 by the loss of mass 122 (C_8_H_10_O) which is proposed to be the next section of the ring (see Scheme 2). Product ion *m*/*z* 464 can also lose mass 56 to generate product ion *m*/*z* 408. It is likely that the 56 Da fragment is located adjacent to the propionyl group, as accurate mass measurements indicate its formula to be C_3_H_4_O (see Table S3). Product ion *m*/*z* 408 can then fragment further by loss of the desosamine sugar to generate product ion *m*/*z* 233. The final ion observed is the protonated desosamine at *m*/*z* 158. MS^3^ analysis of *m*/*z* 464 (Figure 3b) shows four product ions. The only newly observed ion is *m*/*z* 420. MS^4^ analysis of the ion with *m*/*z* 408 (Figure 3c) produced new product ions *m*/*z* 286 and 272. Product ion *m*/*z* 233 is also present as expected, along with *m*/*z* 176, which is the desosamine-protonated ion (see Scheme 2). MS^3^ analysis of the product ion *m*/*z* 558 (see Figure S5b) revealed no new product ions; however, MS^4^ analysis of the *m*/*z* 500 ion (see Figure S6a) produced a product ion at *m*/*z* 482, which indicates that H_2_O elimination is still possible following the first ring cleavage. The MS^4^ analysis of product ion *m*/*z* 342 (see Figure S6b) produced three product ions, *m*/*z* 298, 158 and 116. The structure of product ion 298 is proposed in Scheme 2. Finally, the MS^5^ analysis of product ion *m*/*z* 298 only produced *m*/*z* 158. Scheme 2 shows the sub fragmentation of *m*/*z* 522, with structures proposed for all the product ions discussed above.

### 2.2. Structural Elucidation of the Erythromycin Analogues

ESI-MS of the commercial EryA sample (see Figure S4 and Table S2) indicated there were at least three other components present at low concentrations at *m*/*z* 748, 720 and 718. It is assumed that these are co-metabolites present due to the natural preparation of the sample, and these were initially labelled EryX, EryY and EryZ respectively (see Table S2). The direct infusion MS^n^ fragmentation analysis of each of these co-metabolites was performed and the results compared to the EryA fragmentation above to elucidate their structures; their identification was proposed.

#### 2.2.1. MS^n^ Analysis of EryX (*m*/*z* 748)

**Figure 4:**
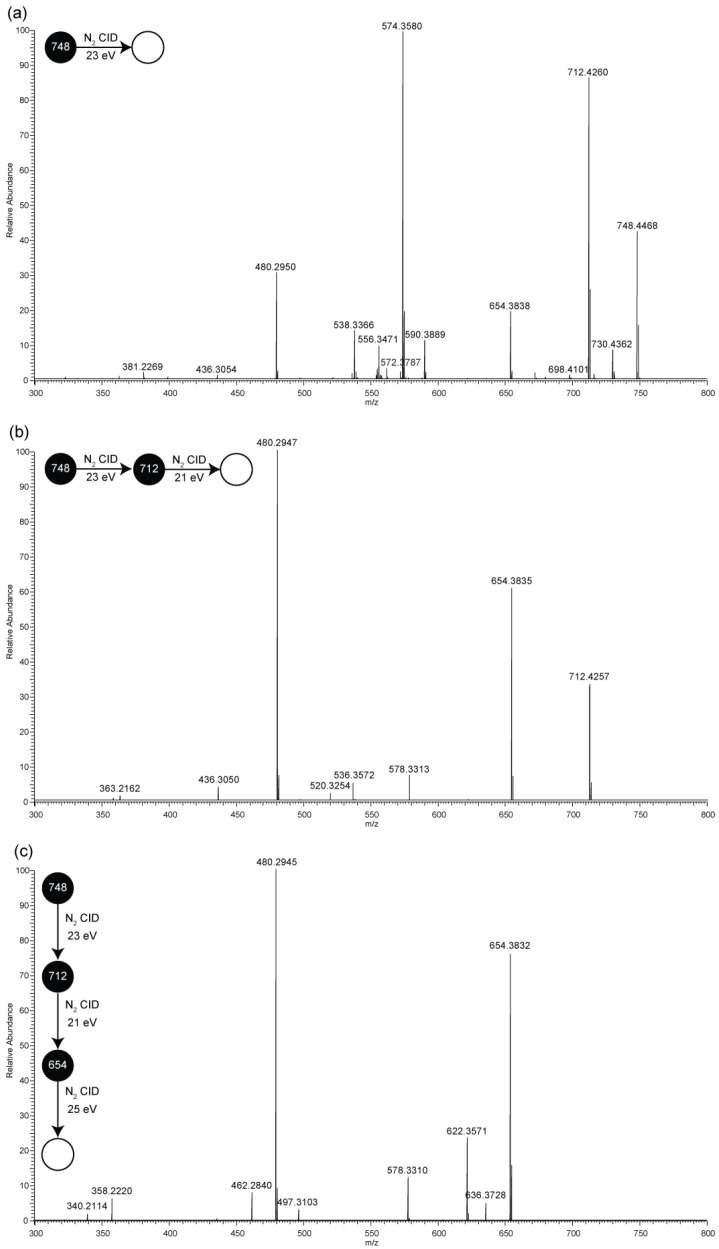
ESI-CID-MS^n^ spectra of co-metabolite EryX. Spectrum (**a**) is the MS^2^ of *m*/*z* 748, spectrum (**b**) is the MS^3^ of *m*/*z* 712 and spectrum (**c**) is the MS^4^ of *m*/*z* 654. The collision energies and MS^n^ sequences are displayed on the spectra.

Co-metabolite EryX has a measured *m*/*z* of 748.4474 which corresponds to the formula C_37_H_66_NO_14_^+^ to 0.5 ppm mass accuracy. This is one oxygen more and two hydrogens less than EryA and is probably due to a single oxidation. Over 20 product ions are clearly present in the MS/MS spectrum of the [M + H]^+^ of EryX (PI *m*/*z* 748), as shown in Figure 4a, while all accurate masses are in Table S4. This is considerably more than the number of ions observed in the MS/MS of EryA (Figure 2a) and suggests that either this co-metabolite fragments much easier or it is a mixture of isomers. The ions at *m*/*z* 730 and 712 are due to the successive losses of water from the PI. The MS/MS spectrum of EryA also has the formation of product ions due to two water losses. This suggests that EryX initially undergoes the same fragmentation as EryA. However, EryX has an additional ion at *m*/*z* 698 matching to the loss of methanol from *m*/*z* 730. This is the first deviation from the fragmentation route followed by EryA and suggests that there is a new low-intensity route following the loss of water due to the loss of methanol. This could be a loss from the cladinose sugar moiety (C-3″) which does not occur with EryA, suggesting that the structural alternation between EryA and one of isomers of EryX is stabilising this part of the structure, allowing for this newly observed neutral loss.

The product ion at *m*/*z* 654 is due to loss of two waters and the 58 Da propionyl starter unit, as confirmed by MS^3^ analysis of the ion with *m*/*z* 712 (Figure 4b). This shows that with EryX, the loss of the two hydroxyls is also in competition with the loss of cladinose as well as with the loss of the 58 Da propionyl starter group. The ion with *m*/*z* 574 occurs due to a loss of 174 Da, corresponding to the cladinose sugar and the oxygen connected to it at C-3. The product ions at *m*/*z* 556 and 538 are due to the losses of water from *m*/*z* 574. Product ion *m*/*z* 538 can then go on to produce product ion *m*/*z* 480 by loss of the propionyl starter unit. The MS^4^ of *m*/*z* 654 (Figure 4c) shows product ions at *m*/*z* 636 and 622 by the loss of water or methanol respectively and the ion at *m*/*z* 497 is due to the loss of the desosamine moiety. The product ion at *m*/*z* 590 is due to the loss of cladinose form the PI and this ion then has a water loss to generate *m*/*z* 572 and a loss of CO to generate the ion at *m*/*z* 562. This is a new route not observed with any erythromycin analogues before and suggests that this specific isomer has an aldehyde group. The other ions in the spectrum are, as previously observed, apart from *m*/*z* 462 which is not observed in the MS^4^ analysis of *m*/*z* 480 (see below) and so cannot be due to the loss of water from this ion. It would appear that *m*/*z* 462 is produced by an alternative route, possibly via *m*/*z* 636, but this cannot be ascertained from the current data. Finally, the MS^4^ analysis of the *m*/*z* 480 (Figure S7) produces just two product ions. One at low intensity (*m*/*z* 323) due to loss of the desosamine moiety and one at *m*/*z* 158 which is the protonated desosamine ion, as observed previously. There is no ring fragmentation observed from this ion.

The presence of the 158 Da loss (for example, from *m*/*z* 748 to 590) and 174 Da losses (for example, from *m*/*z* 748 to 574, *m*/*z* 712 to 538 and *m*/*z* 654 to 480—see Table S4) in the EryX fragmentation pathway indicates that the cladinose sugar is present and its structure is unmodified. Similarly, the presence of an unmodified desosamine moiety is evidenced by losses of 157 Da (for example from *m*/*z* 654 to 497, *m*/*z* 520 to 363, and *m*/*z* 480 to 323—see Table S4) and by the presence of the product ion with *m*/*z* 158 (which is protonated desosamine). Therefore, the 14 Da difference between EryA and one of the EryX isomers must be due to a modification on the macrolide ring. It is assumed that the loss of 58 Da from *m*/*z* 712 to 654, *m*/*z* 578 to 520 and *m*/*z* 538 to 480 shows that the starter unit is still propionate. The loss of methanol from *m*/*z* 730 to 698 is a new route not observed with EryA and this suggests the structural difference must be in the vicinity of the cladinose sugar.

**Figure 5:**
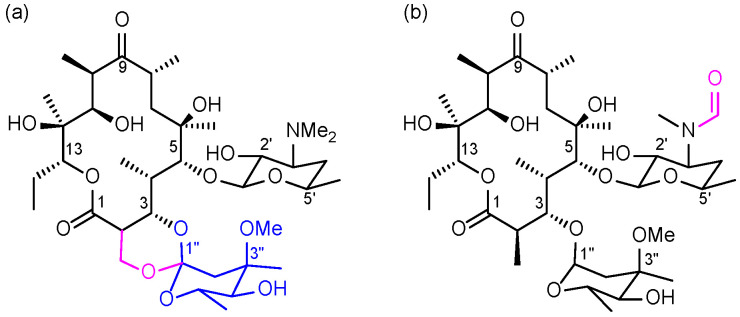
The proposed structures of the two isomeric analogues of erythromycin labelled as EryX1 and EryX2. Structure (**a**) is EryX1 which matches the structure of erythromycin E (EryE). The main difference in the structure is that EryE has an additional ester linkage connecting the cladinose sugar (shown in blue) to the C-2 position of the macrolide ring (shown in pink). Structure (**b**) is EryX2 which matches the structure of erythromycin EP Impurity L (EryEPL). This impurity has a methylated N-formyl group in place of the dimethylamine on the desosamine sugar (shown in pink).

The MS^4^ analysis of the ion with *m*/*z* 654 revealed a new product ion with *m*/*z* 462 that occurs due to an additional loss of hydroxyl. This third hydroxyl loss occurs after the first two. This pattern is also present in the EryA fragmentation, where the lost cladinose is replaced by a hydroxyl at C-3 which is subsequently removed. However, we have already identified the simultaneous loss of the cladinose and glycosidic oxygen in EryX as the ion with *m*/*z* 574. This suggests that there could be two oxygens that bind the cladinose to the lactone ring, which are exposed and removed following the loss of the cladinose. Erythromycin E (EryE) is an erythromycin analogue which matches this description, containing two oxygens connected to the C-2 and C-3 positions of the lactone ring which are bound to the cladinose sugar moiety (see Figure 5). EryE matches the mass of EryX, and as such it has been determined that EryE is one of the co-metabolites with *m*/*z* 748. The second isomer has a loss of CO but otherwise seems to fragment like EryA, which is suggested to be erythromycin EP Impurity L (EryEPL), present in some preparations of erythromycin—see Figure 5 for structures of both isomers. Figure S9 shows the fragmentation tree for the two EryX isomers.

#### 2.2.2. MS^n^ Analysis of EryY (*m*/*z* 720)

**Figure 6:**
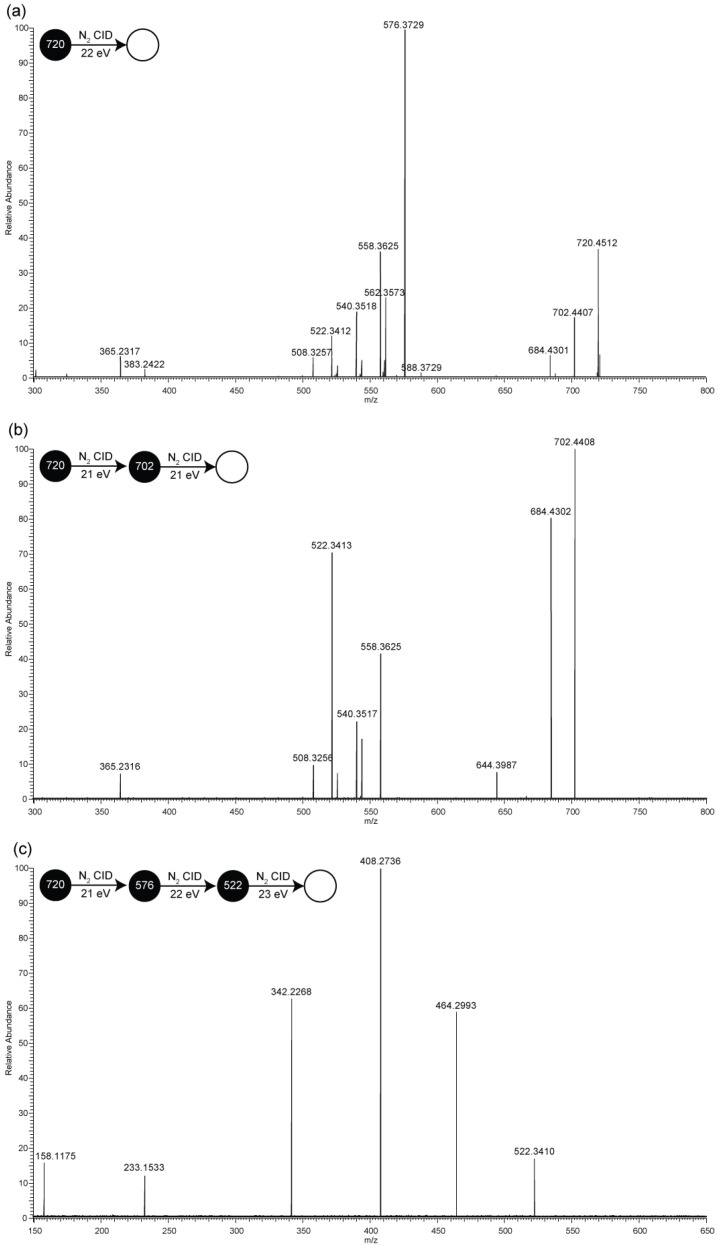
ESI-CID-MS^n^ spectra of co-metabolite EryY. Spectrum (**a**) is the MS^2^ of *m*/*z* 720, spectrum (**b**) is the MS^3^ of *m*/*z* 702 and spectrum (**c**) is the MS^4^ of *m*/*z* 522. The collision energies and MS^n^ sequences are displayed on the spectra.

Co-metabolite EryY has a measured *m*/*z* of 720.4525 which corresponds to the formula C_36_H_66_NO_13_^+^ to 0.5 ppm mass accuracy. This is one CH_2_ less than EryA. This is probably a demethylation. Over 24 product ions are clearly present in the MS/MS spectrum of the [M + H]^+^ of EryY (PI *m*/*z* 720), as shown in Figure 6a and Table S5. There are initially two losses of water to generate product ions at *m*/*z* 702 and 684. The base peak in the spectrum is for the product ion with *m*/*z* 576, due to a loss of 144 Da, which replaces the 158 Da loss with EryA (loss of cladinose). This is followed by three losses of water to generate product ions at *m*/*z* 558, 540 and 522. These are analogues to the fragmentation of EryA after the loss of cladinose. This indicates that the structural alternation in EryY is in the cladinose sugar moiety. It is proposed that the cladinose has been replaced by a mycarose, which would correspond to the 14 Da difference between EryA and EryY. Examination of the MS^3^ of *m*/*z* 702 (Figure 6b and Table S5) supports that there is now a new product ion at *m*/*z* 644 due to the loss of 58 Da matching to the loss of the propionate starter group. Finally, the MS^4^ spectrum of *m*/*z* 522 (Figure 6c and Table S5) is almost identical to the MS^3^ spectrum of 522 for EryA (Figure 3a). The only differences are in the intensity of the ions. This proves that the structure of the macrolide ring is the same between EryA and EryY.

**Figure 7:**
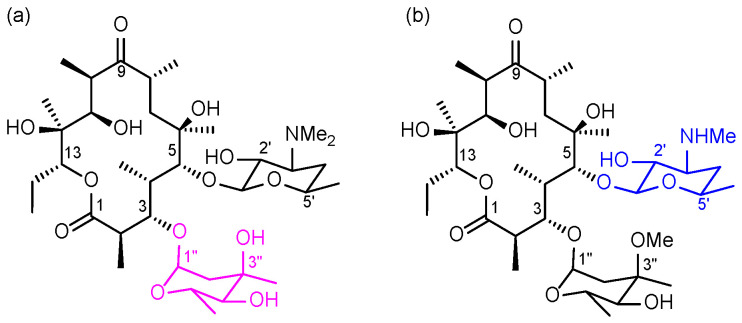
The proposed structures of two isomeric analogues of the co-metabolite EryY. Structure (**a**) matches erythromycin C (EryC), with the main difference in the structure being that EryC contains a mycarose sugar on carbon C-3 of the macrolide ring (shown in pink). Structure (**b**) matches N-demethyl-erythromycin A, with the main difference being demethylation of the amine of the desosamine moiety (shown in blue).

However, there is also a set of product ions at *m*/*z* 562, 544, 526 and 508 (see Figure 6a and Table S5) which correspond to successive water losses after the loss of mass 158. This would suggest that *m*/*z* 720 in the sample is a mixture of two isomers both with a CH_2_ less than EryA. One of these is with mycarose in place of cladinose and the other still shows losses of cladinose (loss of 158 Da) and so must have the CH_2_ difference elsewhere in the structure. After the cladinose losses there is a series of product ions and *m*/*z* 486, 468 and 450 which correspond to the loss of mass 58 from *m*/*z* 544, 526, and 508. The accurate masses (see Table S5) show that the formula of this loss is C_3_H_6_O (propionate) which suggests that this isomer also has the same macrolide ring as EryA and the loss of 158 locates the structural alternation to the desosamine sugar moiety. This contrasts with the paper by Govaerts et al. [37] in which a 720 isomer was due to a starter acid variation with a loss of mass 44 (for ethanoate) in place of the loss of mass 58 (for propionate). Examination of the MS/MS spectra shows that there are very low-intensity ions at *m*/*z* 307 and 289 (separated by a water loss) see Table S5. These are due to the loss of mass 143, and the accurate mass analysis confirms the formula of this loss to be C_7_H_13_NO_2_, which corresponds to N-demethyl-desosamine. This means that the second isomer of Ery720 is N-demethyl EryA (see Table S1). With all these factors considered, the structures of the two isomers of the EryY have been proposed (see Figure 7), with the fragmentation tree shown in Figure S10.

#### 2.2.3. MS^n^ Analysis of EryZ (*m*/*z* 718)

**Figure 8:**
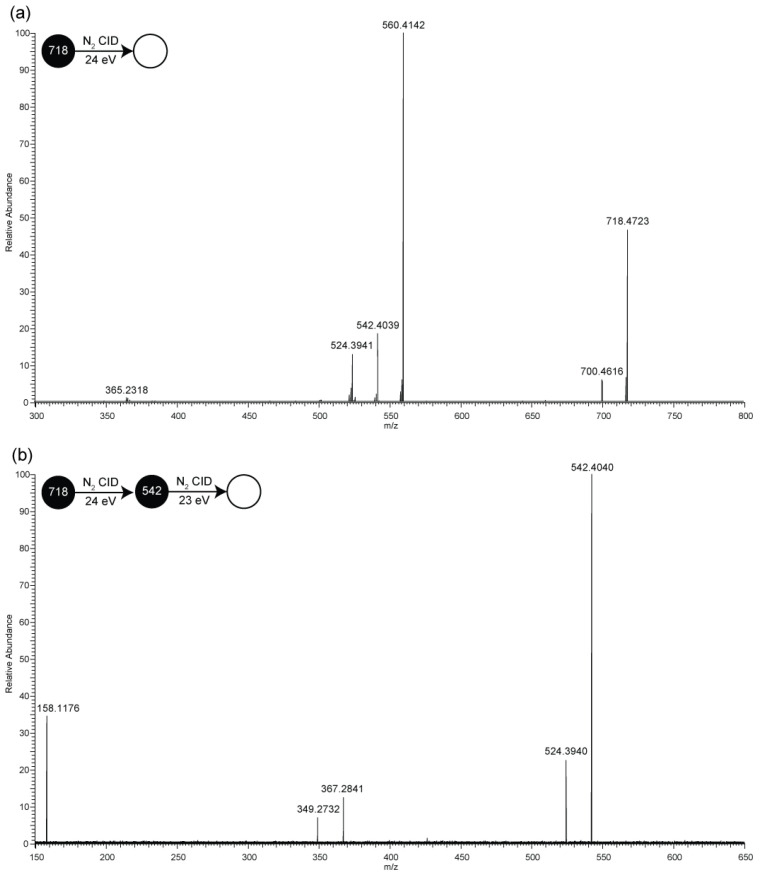
ESI-CID-MS^n^ spectra of co-metabolite EryZ. Spectrum (**a**) is the MS^2^ of *m*/*z* 718 and spectrum (**b**) is the MS^3^ of *m*/*z* 542. The collision energies and MS^n^ sequences are displayed on the spectra.

Co-metabolite EryZ has a measured *m*/*z* of 718.4733 which corresponds to the formula C_37_H_68_NO_12_^+^ to 0.5 ppm mass accuracy. This is one oxygen less than EryA and is probably due to a reduction. There are six product ions observed in the ESI-MS/MS analysis of the [M + H]^+^ ion of EryZ (PI *m*/*z* 718, see Figure 8). The product ion *m*/*z* 700 is due to loss of one hydroxyl. There is no second hydroxyl loss which contrasts to the fragmentation of EryA. Loss of cladinose results in the product ion with *m*/*z* 560 followed by two water losses to *m*/*z* 542 and 524. The loss of the cladinose moiety enables the loss of a new hydroxyl connected to the C-3 position of the lactone ring in the same way as for EryA. This fragmentation sequence is confirmed by the MS^3^ analysis of the ion with *m*/*z* 542 (Figure 8b). The loss of a hydroxyl and cladinose results in the ion with *m*/*z* 524. There is no third loss of hydroxyl, which indicates that either the C-11 or C-12 hydroxyl is missing from the lactone ring. The product ions at *m*/*z* 367 and 349 are related by a water loss and are due to loss of desosamine from *m*/*z* 524. There are no losses of the started group, which suggests that the alteration in the macrolide ring is in the vicinity of C-13, and so it is proposed that the C-12 hydroxy is the one that is missing. This spectrum also confirms the presence of the *m*/*z* 158, which is the protonated desosamine ion.

**Figure 9:**
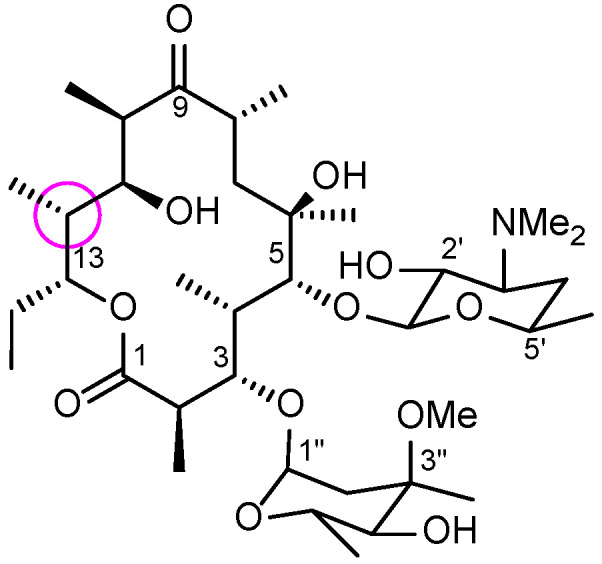
The proposed structure of the EryZ co-metabolite which matches the structure of erythromycin B (EryB). The main difference in the structure is that EryB has a missing hydroxy group on carbon C-12 of the macrolide ring (circled in pink).

It is therefore proposed that the identity of EryZ is the co-metabolite erythromycin B (EryB—see Figure 9). Here, the hydroxyl group at C-13 is replaced by hydrogen, resulting in EryB having a mass 14 Da less than EryA.

### 2.3. Identification of the Co-Metabolites of EryA by LC-MS/MS

**Figure 10:**
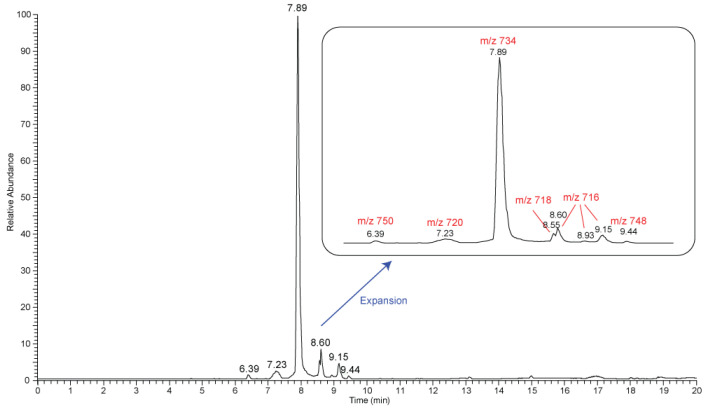
LC-MS chromatogram of a commercial sample of erythromycin. The boxed insert shows an expansion of the region of interest where the erythromycin co-metabolites elute with peaks labelled by the molecular weight in red.

LC-MS analysis of the commercial sample of erythromycin (Figure 10) shows the presence of the co-metabolites as expected. The main compounds occur at *m*/*z* 750 (6.39 min), 748 (9.44 min), 734 (7.89 min, EryA), 720 (broad peak from 7 to 7.6 min), 718 (8.55 min) and 716 (3 peaks at 8.60, 8.93 and 9.15 min). This would suggest that there are least eight co-metabolites of erythromycin present, which is more than expected, and so LC-MS/MS analysis was performed in an order to try to identify them using the previous knowledge of the fragmentation of EryA, EryB, EryC and EryE from the direct infusion MS^n^ analysis discussed in detail above.

**Figure 11:**
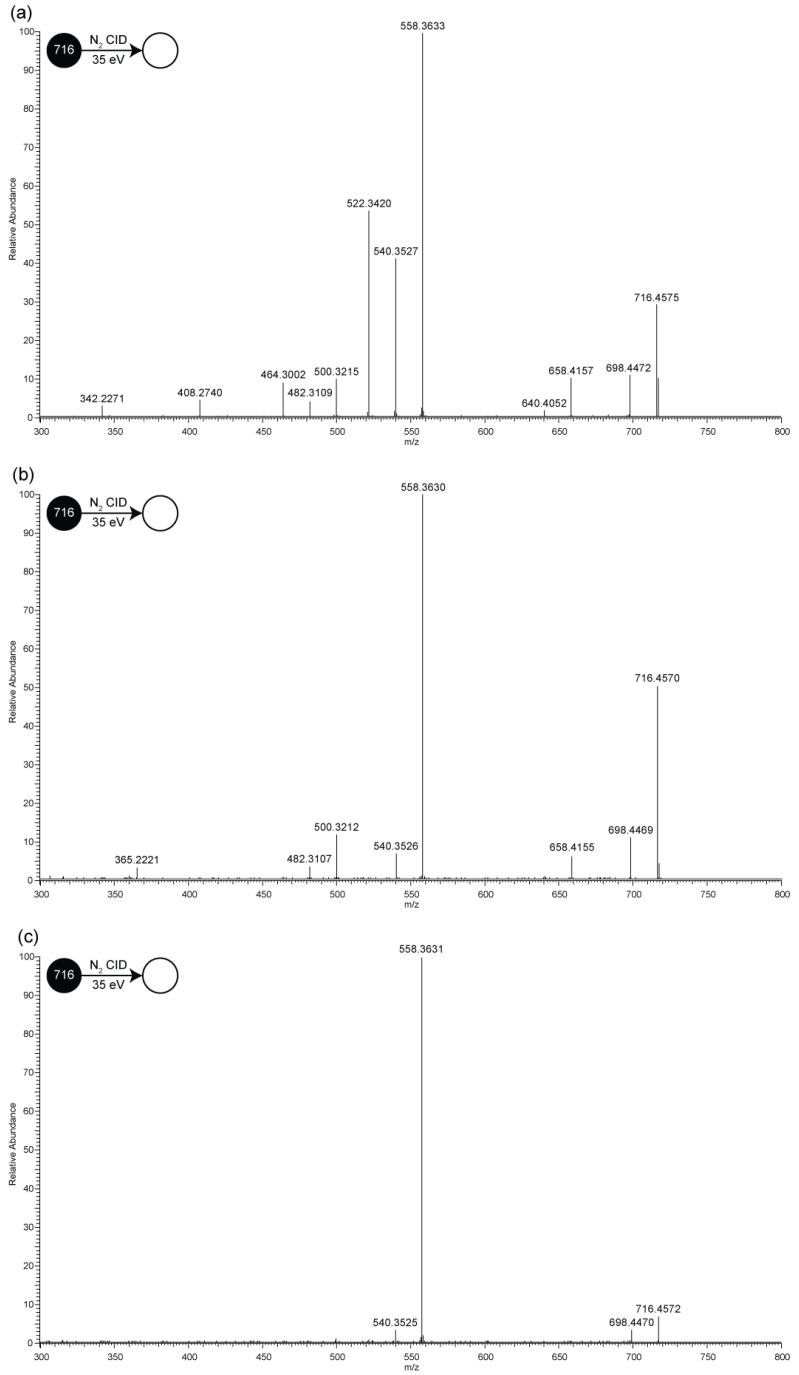
LC-MS/MS spectra of the three co-metabolite isomers with [M + H]^+^ at *m*/*z* 716. Spectrum (**a**) is for isomer Ery716A eluting at 8.55 to 8.69 min, spectrum (**b**) is for isomer Ery716B eluting at 8.89 to 8.96 min and spectrum (**c**) is for isomer Ery716C eluting at 9.08 to 9.20 min. The collision energies are displayed on the spectra.

The three isomers at *m*/*z* 716 (labelled Ery716A, Ery716B and Ery716C respectively) were considered first, and their LC-MS/MS spectra are shown in Figure 11. The measured masses of the three isomeric Ery716 co-metabolites match to C_37_H_66_NO_12_^+^ to approximately 1 ppm mass accuracy. This corresponds to a loss of water compared to EryA—this is assumed to be dehydration. One of the key difficulties in isomer separation and elucidation by LC-MS/MS is that the isomers tend to have very similar fragmentation with only subtle differences—this is clearly seen in the three spectra in Figure 11. However, the main difficulty is that due to chromatographic time constraints and the lack of data points across a chromatographic peak, you can only perform MS/MS with online HPLC. This means that you lose access to sequential mass spectrometry and the ability to ‘drill down’ into the structure of product ions. The three Ery716 co-metabolites correspond to the dehydration of EryA and this can happen at any of the hydroxy groups. Two fully elucidated structures of dehydrated EryA are known in the literature [14,15,16] (see Figure S2). These are bi- and tri-cyclic structures. It is suspected that a third dehydration could occur from either of the sugar moieties attached to the macrolide ring.

In the MS/MS spectrum of Ery716A (Figure 11a), product ion *m*/*z* 698 results from a single water loss, and the base peak (*m*/*z* 558) corresponds to loss of 158 Da, which is due to loss of cladinose. There are then two water losses to generate *m*/*z* 540 and 522. There are also product ions at *m*/*z* 658 and 640 which are proposed to be due to the loss of the propionate starter group (loss of 58 Da) from the PI and *m*/*z* 698 respectively. The loss of 58 Da also occurs from product ions *m*/*z* 558, 540 and 522 to generate product ions *m*/*z* 500, 482 and 464. These fragments are analogous to EryA but with one less water loss. They prove that cladinose is intact and the starter acid is still propionate which can still be lost. This suggests that the hydroxy group on C-12 is present. There is no indication of a loss of the desosamine moiety and so it is proposed that isomer Ery716A results from dehydration across C-1′ and C-2′ which prevents the loss of desosamine. This structure has not previously been published in the literature.

Figure 11b shows the MS/MS spectrum of Ery716B. Product ions *m*/*z* 698, 658, 558, 540, 500, and 482 are proposed to be generated by analogous routes to the same ions generated for Ery716A. However, there is a missing water loss—i.e., there are no ions observed at *m*/*z* 640, 522 or 464. This would suggest that one of the macrolide hydroxy groups is missing, but because the loss of the propionate starter group is still observed (loss of 58 Da from *m*/*z* 716 to 658, 558 to 500 and 540 to 482), this would suggest that C-12 is present. Additionally, there is a low-intensity product ion at *m*/*z* 365 which is presumably due to the loss of desosamine (loss of 175 Da) from *m*/*z* 540, which further locates the site of the dehydration to the macrolide ring. These factors, when combined, lead to the assignment of the structure of this co-metabolite as being the structure shown in Figure S2a.

For Figure 11c, the MS/MS of Ery716c, there are only three product ions observed, which is a significant reduction in fragmentation indicative of reduced molecular confirmational degrees of freedom, and so it is proposed that this is the spiroketal form of dehydrated EryA, which is the structure shown in Figure S2b.

The co-metabolites of erythromycin occurring at *m*/*z* 718, 720 and 748 are labelled Ery718, Ery720 and Ery748 respectively. Ery718 has a measured *m*/*z* of 718.4731 which corresponds to C_37_H_68_NO_12_^+^ to 0.7 ppm. Ery720 has a measured *m*/*z* of 720.4511, which corresponds to the formula C_36_H_66_NO_13_^+^ to 2.5 ppm mass accuracy, and finally Ery748 has a measured *m*/*z* of 748.4463, which corresponds to the formula C_37_H_66_NO_14_^+^ to 2.0 ppm mass accuracy. The HPLC-ESI-CID-MS/MS spectra of these three co-metabolites are found in Figure S8, with detailed accurate masses listed in Table S9. When comparing these spectra to the direct infusion MS/MS spectra for the co-metabolites, it can be seen that they are almost identical, with most of the product ions being in common with only differences in the peak intensities. This is due to the HPLC-MS/MS analysis being conducted at a higher collision energy (35 eV), leading to an overall increase in fragmentation and reduction in the intensity of the higher massed product ions. This leads to the following assignments: Ery718 as erythromycin B, Ery720 as being erythromycin C (the other 720 isomer is at very low intensity in the chromatogram and no MS/MS spectrum was obtained) and Ery748 is erythromycin E.

**Figure 12:**
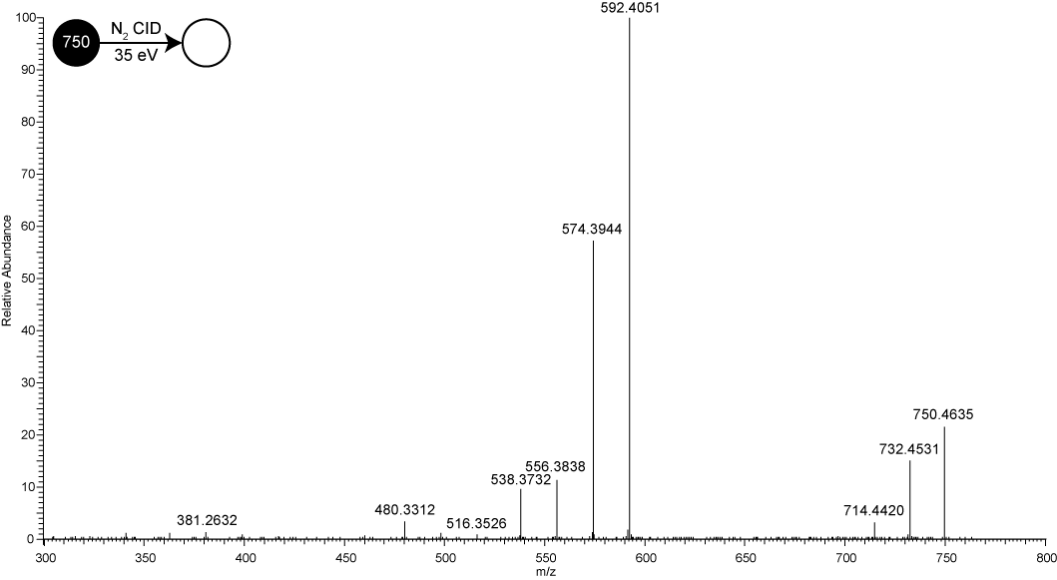
The LC-MS/MS spectrum of the co-metabolite with [M + H]^+^ at *m*/*z* 750 (designated Ery750) eluting at 6.33 to 6.49 min. The collision energy is displayed on the spectrum.

**Figure 13:**
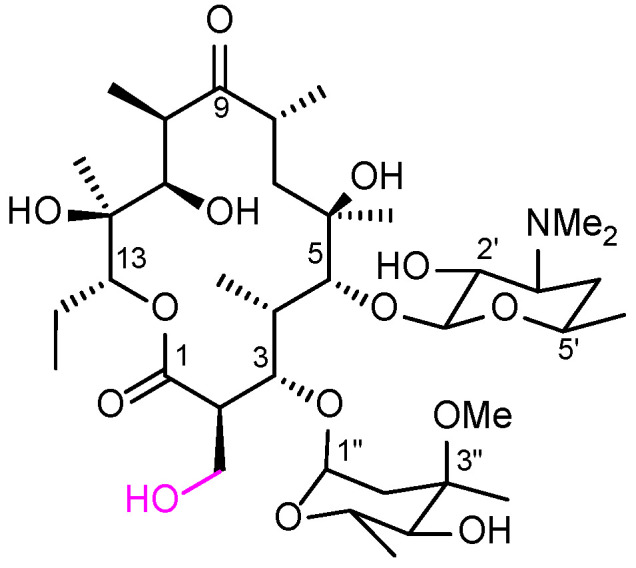
The proposed structure of the Ery750 co-metabolite which matches the structure of erythromycin F (EryF). The main difference in the structure is that EryF has an extra hydroxy group attached to the methyl on carbon C-2 of the macrolide ring (shown in pink).

Finally, we need to consider the *m*/*z* 750 co-metabolite which has been labelled Ery750 with its HPLC-MS/MS spectrum shown in Figure 12. The measured mass of Ery750 is 750.4635, which matches less than 0.20 ppm to C_37_H_68_NO_14_^+^, which is one oxygen more the EryA. The HPLC-MS/MS spectrum is very similar to the MS/MS mass spectrum of EryA but with the product ions occurring 16 Da higher. This would suggest that the extra hydroxy group is not in a position that affects the fragmentation, and not on either of the sugar moieties. This rules out a lot of the carbons around the macrolide ring and so it is proposed that this co-metabolite is erythromycin F with the additional OH group being located on the methyl group of C-2—see Figure 13 for the structure.

## 3. Materials and Methods

Erythromycin A (E077) was obtained from Sigma-Aldrich (Gillingham, UK) and all solvents (HPLC gradient grade) were obtained from Fisher Scientific (Loughborough, UK). All mass spectral analyses were performed on an Orbitrap Elite (Thermo Scientific, Hemel Hempstead, UK) by positive ion MS using a heated electrospray ionisation (HESI) source with a typical *m*/*z* range from 100 to 800. ESI was performed at a source voltage of 3 kV, source temperature of 350 °C and a capillary temperature of 275 °C. For direct infusion analyses, solutions were introduced into the instrument via a syringe pump at a flow rate of 5 mL/minute. Sequential (MS^n^) experiments were recorded at a resolution of 60,000 on isolated precursor ions (2 Da window) using collision-induced dissociation (CID) with high-purity (oxygen-free) N_2_ collision gas at the normalised collision energy indicated on the spectra. HPLC and HPLC in-line tandem mass spectrometry (HPLC-MS/MS) analysis was performed on the Orbitrap Elite using a Dionex ultimate 3000RS UPLC system (Thermo Scientific, Hemel Hempstead, UK). Chromatography was performed using a Waters (Wilmslow, UK) Acquity UPLC BEH C18 column (2.10 × 100 mm, 1.7 µm ID). Mobile phase A consisted of water with 0.1% formic acid. Mobile phase B consisted of acetonitrile with 0.1% formic acid. UHPLC gradients ran from 5% B to 95% B over 20 min. The HPLC-MS/MS analysis was carried out in data-dependent mode at 35 eV normalised collision energy using CID in the Ion-Trap component of the Orbitrap Elite mass spectrometer with the product ions mass measured in the Orbitrap component at a resolution of 15,000. For all analyses, the Orbitrap was calibrated with an external calibration using Pierce LTQ ESI Positive Ion Calibration Solution (88323) (Thermo Scientific, Hemel Hempstead, UK) using the built-in auto tune and calibration algorithms.

## 4. Conclusions

For LC-MS/MS to be an effective technique for the structural elucidation of novel natural products, natural co-metabolites or decomposition products, the fragmentation of the original compound must first be fully elucidated and understood. In order to elucidate the full fragmentation schemes for natural products, ultra-high resolution accurate-mass sequential mass spectrometry is required—typically using FT-ICR or Orbitrap instrumentation. This is to be able to ‘drill down’ into target structures and obtain unequivocal formulae for all fragment ions observed. In the case of the macrocyclic polyketide, erythromycin A, there remain large gaps in the understanding of its fragmentation routes in the literature.

However, there are a few key observations in the existing literature [30,31,32]. Firstly, the order of the sugars lost is always the C-3 (cladinose with EryA) competing with losses of water, followed by loss at C-5 (desosamine with EryA) competing with losses of the starter group. This second observation is that the first loss from the macrolide ring was always the starter group and by changing it by biosynthetic means, this would change the mass of this loss observed. Thirdly, the first loss of water was via an internal nucleophilic attack from the hydroxy group at C-6 onto the carbonyl at C-9 leading to internal cyclisation. This process is aided by protonation of the carbonyl group—and was proven by oxygen-18 labelling and the study of the oxime-substituted analogue of EryA (roxithromycin) which always loses the oxime group as the first loss in place of water. This observation was supported by the existing literature which elucidated the structure of dehydrated EryA as having one or two internal cyclisations [14,15,16]. The final observation is that upon MS^n^ analysis of EryB, where the C-13 hydroxyl is absent, the loss of the second hydroxyl group was also absent, which pinpoints the second water loss to be from the C-13 hydroxyl and not the one at C-12. All these existing observations are incorporated into the extended fragmentation mechanisms proposed in this study (see Scheme 1 and Scheme 2).

Using high-resolution accurate-mass ESI-MS^n^ we have been able to propose structures for all product ions of EryA to MS^4^ level. This has enabled us to generate a more useful extended fragmentation scheme. This was then used to assign structures to the co-metabolites present in the commercial sample. Initially, 4 co-metabolites were studied by direct infusion MS/MS and the fragmentation compared to that of EryA. This led to the structural differences being spotted and for us to propose them to be erythromycin B, C, E and erythromycin EP Impurity L. The same sample was then analysed by UHPLC-MS/MS to try to separate possible isomeric components. Using this approach, the presence of erythromycin B, C and E was confirmed by comparing the LC-MS/MS spectra to those obtained by direct infusion. It was also possible to detect and assign structures to several more co-metabolites including 3 isomeric dehydration products (two previously recorded and one not). This was more difficult, as assignment of structure to isomers by MS/MS can be more challenging; however, in this case, the three MS/MS spectra were considerably different. The use of logical deductions enabled the assignment of one of the isomers as being the result of dehydration in the desosamine moiety—this is a previously unpublished structure. The other two isomers were proposed to be the previously reported dehydration products. Additionally, in the UHPLC-MS/MS analysis, the presence of erythromycin F was also determined. These results highlight the power of accurate-mass LC-MS/MS for structural elucidation when an extended fragmentation scheme is available. The structures of all the co-metabolites observed are shown in Figure S11.

## Data Availability

The raw data are available from the University of Bristol data repository, data.bris, at https://data.bris.ac.uk/data/dataset/1z2toohfnv3hj2idy4ox4hsnpi (accessed on 8 March 2026).

## Supplementary Materials

The following supporting information can be downloaded at https://www.mdpi.com/article/10.3390/molecules31060928/s1. Refs. [8,13,14,15,30,31,38] are cited in the Supplementary Materials.

## Abbreviations

The following abbreviations are used in this manuscript:

CID	Collision-Induced Dissociation
Ery	Erythromycin
ESI	Electrospray Ionisation
FT-ICR	Fourier-Transform Ion Cyclotron Resonance
HESI	High Temperature Electrospray Ionisation
HPLC	High Performance Liquid Chromatography
LC	Liquid Chromatography
MS	Mass Spectrometry
MS/MS	Tandem Mass Spectrometry
MS^n^	Sequential Mass Spectrometry
PI	Precursor Ion
PKS	Polyketide Synthase
ppm	Parts per Million
