# Supplementary material for: An Electrospray Sequential Mass Spectrometry Fragmentation Scheme of Erythromycin A and Its Application for the Elucidation of the Structures of Its Natural Co-Metabolites
Source: Molecules. 2026 Mar 11;31(6):928. doi: 10.3390/molecules31060928 (PMC13028665; doi:10.3390/molecules31060928)
Supplement: Supplementary file 1 [file molecules-31-00928-s001.zip › molecules-4151504-supplementary.pdf]

An Electrospray Sequential Mass Spectrometry Fragmentation Scheme of Erythromycin A and Its Application for the Elucidation of the Structures of Its Natural Co-Metabolites

Candy Jiang and Paul J. Gates *

School of Chemistry, University of Bristol, Cantock's Close, Bristol BS8 1TS, UK; candy.jiang@bristol.ac.uk
* Correspondence: paul.gates@bristol.ac.uk; Tel.: +44-(0)117-3317192

Supplementary Information.

Contents:

Figure S1. The generic structure of the Erythromycin class of macrolide.

Table S1. The various substituent groups that make up the erythromycin family of macrolide antibiotic analogues.

Figure S2. The byproducts of the acid-catalysed decomposition of EryA.

Figure S3. The structures of the various erythromycin variants analysed in the starter acid units study.

Figure S4. ESI- MS spectrum of a commercial sample of EryA.

Table S2. The observed *m/z*, formulae, theoretical *m/z*, error (ppm) and ion identification from the MS analysis of EryA.

Figure S5. ESI-CID-MS^n spectra of EryA.

Figure S6. ESI-CID-MS^n spectra of EryA.

Figure S7. ESI-CID-MS^n spectrum of EryX**.**

Figure S8. UHPLC-ESI-CID-MS/MS spectra of Erythromycin co-metabolites.

Figure S9. The fragmentation tree for the ESI-MS^n analysis of EryX ion (PI = *m/z* 748).

Figure S10. The fragmentation tree for the ESI-MS^n analysis of EryY ion (PI = *m/z* 720).

Figure S11. The proposed structures of all the erythromycin co-metabolites and degradation products detected in this study.

Table S3. Table of observed *m/z*, formulae, theoretical *m/z*, error (ppm) and ion identification from the MS^n analysis of $[EryA+H]^+$.

Table S4. Table of observed *m/z*, formulae, theoretical *m/z*, error (ppm) and ion identification from the MS^n analysis of $[EryX+H]^+$.

Table S5. Table of observed *m/z*, formulae, theoretical *m/z*, error (ppm) and ion identification from the MS^n analysis of protonated EryY.

Table S6. Table of observed *m/z*, formulae, theoretical *m/z*, error (ppm) and ion identification from the MS^n analysis of protonated EryZ.

Table S7. Table of observed *m/z*, formulae, theoretical *m/z*, error (ppm) and ion identification from the UHPLC-MS/MS analysis of protonated Ery716 isomers.

Table S8. Table of observed *m/z*, formulae, theoretical *m/z*, error (ppm) and ion identification from the UHPLC-MS/MS analysis of protonated Ery750.

Table S9. Table of observed *m/z*, formulae, theoretical *m/z*, error (ppm) and ion identification from the UHPLC-MS/MS analysis of protonated Ery718, Ery720 and Ery748.

The constituent structures present in a commercial preparation of erythromycin.

The 6 erythromycin analogues which are supposed to be present in commercial samples are designated erythromycin A, B, C, D, E and F (see figure S1 and table S1) [S1]. There is also a desosamine N-demethylation analogue that may be present. Of these, erythromycin A (EryA) possesses the strongest antibacterial properties followed by B, then C and D, then the remaining analogues [S2]. EryA is a 14-membered macrolide, with an erythranolide lactone ring. At the C-5 position of the ring is a glycosidic bond to a D-desosamine sugar moiety and at the C-3 position a L-cladinose sugar moiety is attached.

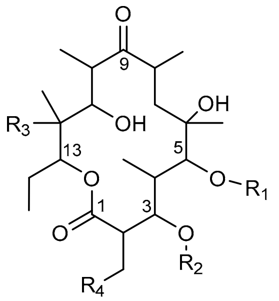

Figure S1. The generic structure of the Erythromycin class of macrolide with the various substituent positions numbered from R_1 to R_4 (see table S1).

Table S1. The various substituent groups that make up the erythromycin family of macrolide antibiotic analogues (see figure S1 for the structures). The sugars are desosamine (Des), N-demethyl-desosamine (NdMeDes), cladinose (Clad) and mycarose (Myc). The formulae and molecular weights (Mw) of the compounds are also indicated.

Designation	R_1	R_2	R_3	R_4	Formula	Mw
Erythromycin A	Des	Clad	-OH	-H	$C_{37}H_{67}NO_{13}$	733
Erythromycin B	Des	Clad	-H	-H	$C_{37}H_{67}NO_{12}$	717
Erythromycin C	Des	Myc	-OH	-H	$C_{36}H_{65}NO_{13}$	719
Erythromycin D	Des	Myc	-H	-H	$C_{36}H_{65}NO_{12}$	703
Erythromycin E	Des	Clad	-OH	-O-	$C_{37}H_{65}NO_{14}$	747
Erythromycin F	Des	Clad	-OH	-OH	$C_{37}H_{67}NO_{14}$	749
N-Dimethyl EA	NdMeDes	Clad	-OH	-H	$C_{36}H_{65}NO_{13}$	719

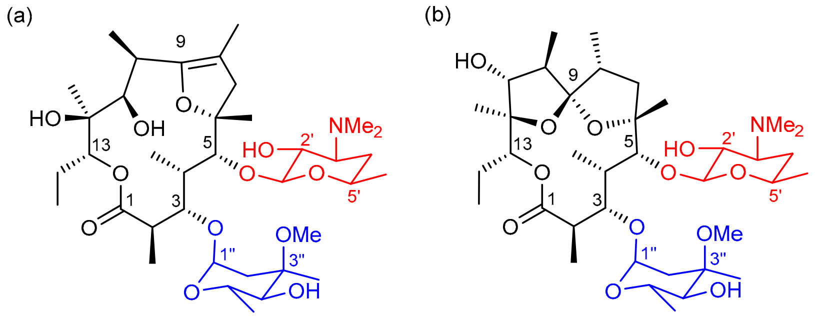

Figure S2. The byproducts of the acid-catalysed decomposition of EryA [S3-S5].

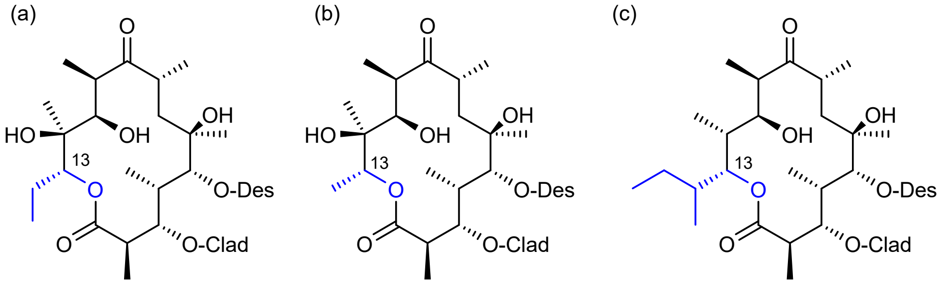

Figure S3. The structures of the various erythromycin variants analysed in the starter acids study, with the starter (including C-13) coloured blue. Structure (a) is EryA (with a propionate starter unit) (b) is Ery1 (with an acetate starter unit) and (c) is Ery2, a variant of erythromycin B with a secondary butyrate starter unit [S6, S7].

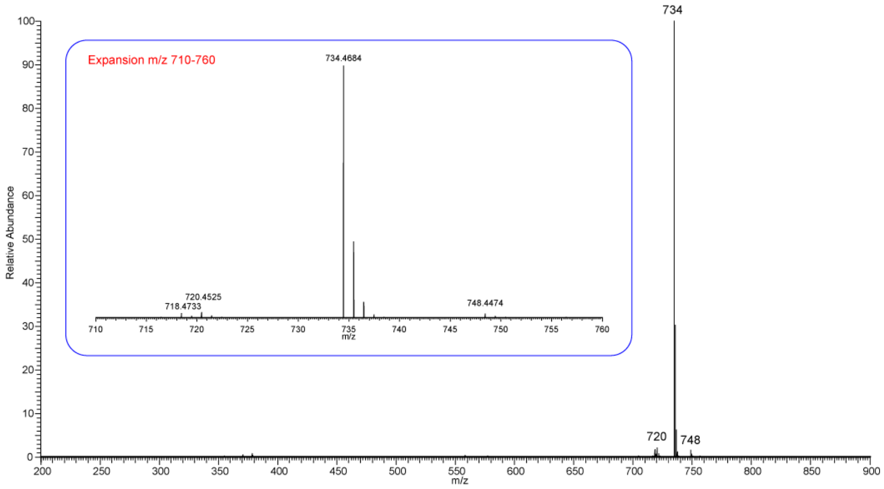

Figure S4. ESI- MS spectrum of a commercial sample of EryA. The boxed insert is an expansion of *m/z* 710 to 760 showing the co-metabolites present in the sample.

Table S2. The observed *m/z*, formulae, theoretical *m/z*, error (ppm) and ion identification from the MS analysis of EryA (figure S4).

**Observed *m/z***	**Formula**	**Theoretical *m/z***	**Error (ppm)**	**Identification**
748.4474	$C_{37}H_{66}NO_{14}^{+}$	748.4478	0.53	Co-metabolite 'EryX'
734.4684	$C_{37}H_{68}NO_{13}^{+}$	734.4685	0.14	Erythromycin A
720.4525	$C_{36}H_{66}NO_{13}^{+}$	720.4529	0.56	Co-metabolite 'EryY'
718.4733	$C_{37}H_{68}NO_{12}^{+}$	718.4736	0.42	Co-metabolite 'EryZ'

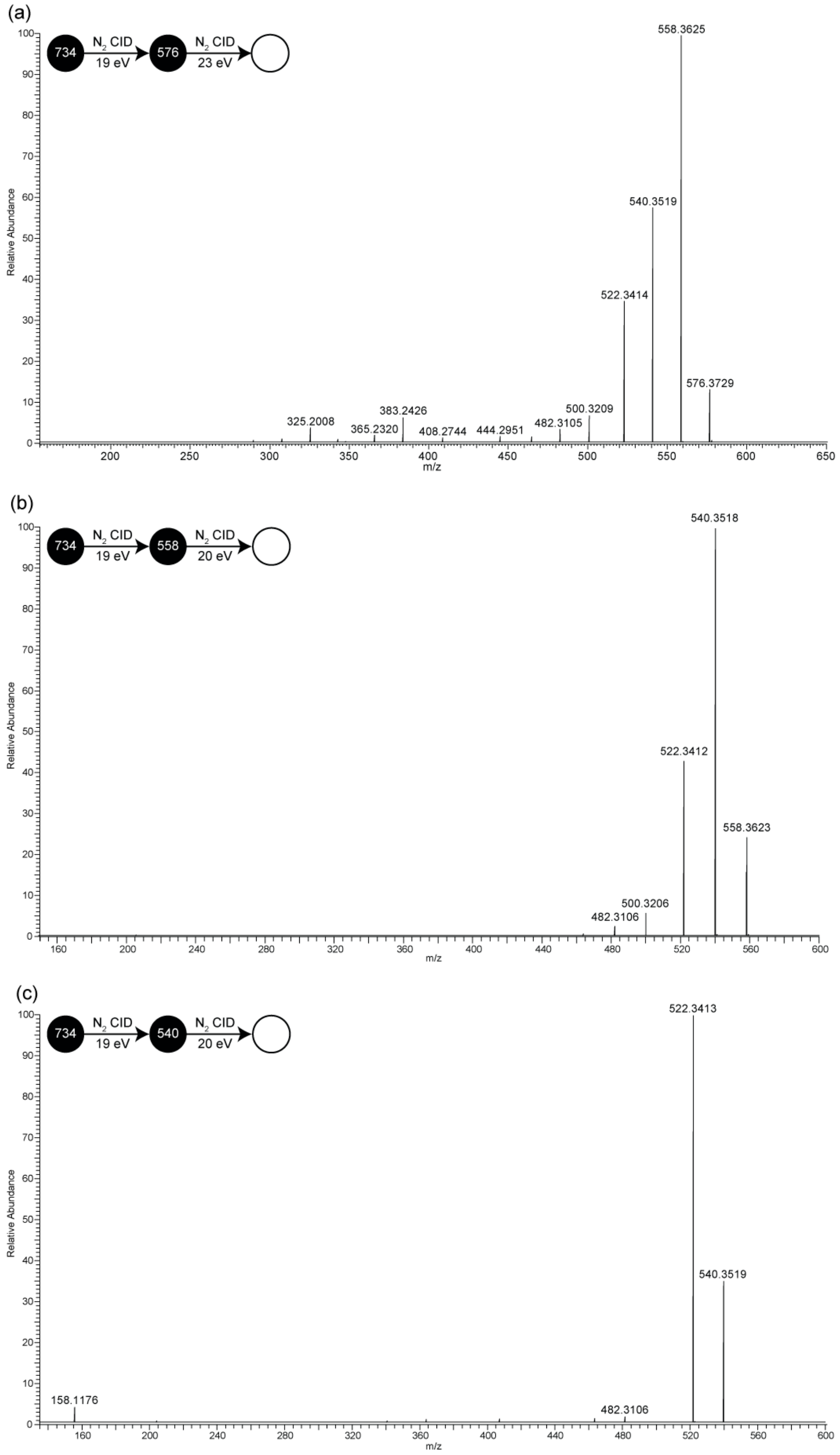

Figure S5. ESI-CID-MS^n spectra of EryA. Spectrum (a) is the MS^3 of *m/z* 576, spectrum (b) is the MS^3 of *m/z* 558 and spectrum (c) is the MS^3 of *m/z* 540. The collision energies and MS^n sequences are displayed on the spectra.

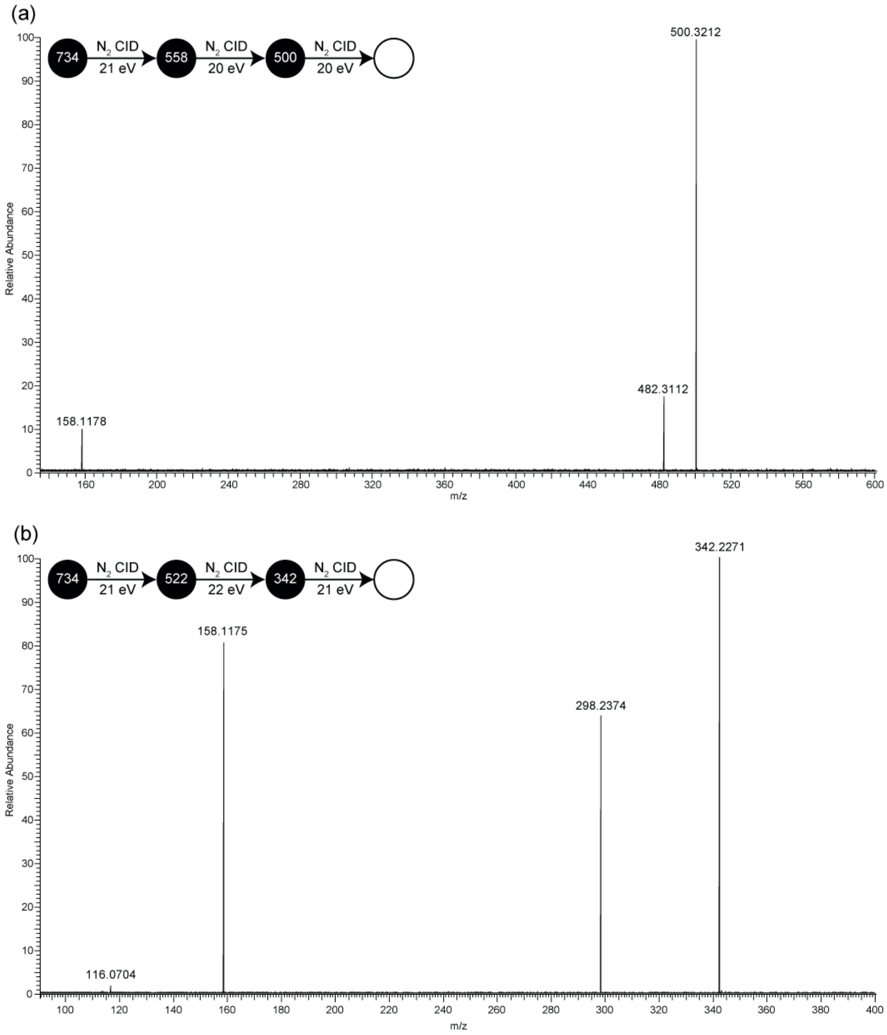

Figure S6. ESI-CID-MS^n spectra of EryA. Spectrum (a) is the MS^4 of *m/z* 500, spectrum (b) is the MS^4 of *m/z* 342. The collision energies and MS^n sequences are displayed on the spectra.

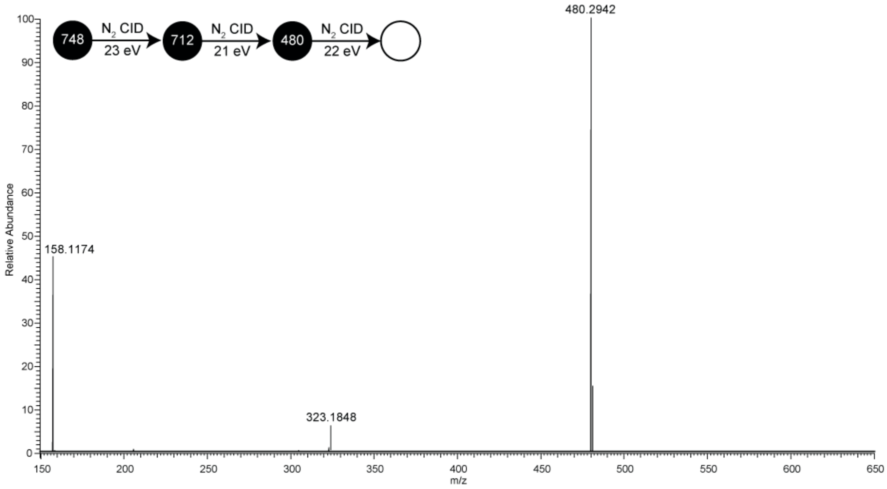

Figure S7. ESI-CID-MS^n spectrum of EryX. The spectrum is the MS^4 of *m/z* 480. The collision energy and MS^n sequences are displayed on the spectra.

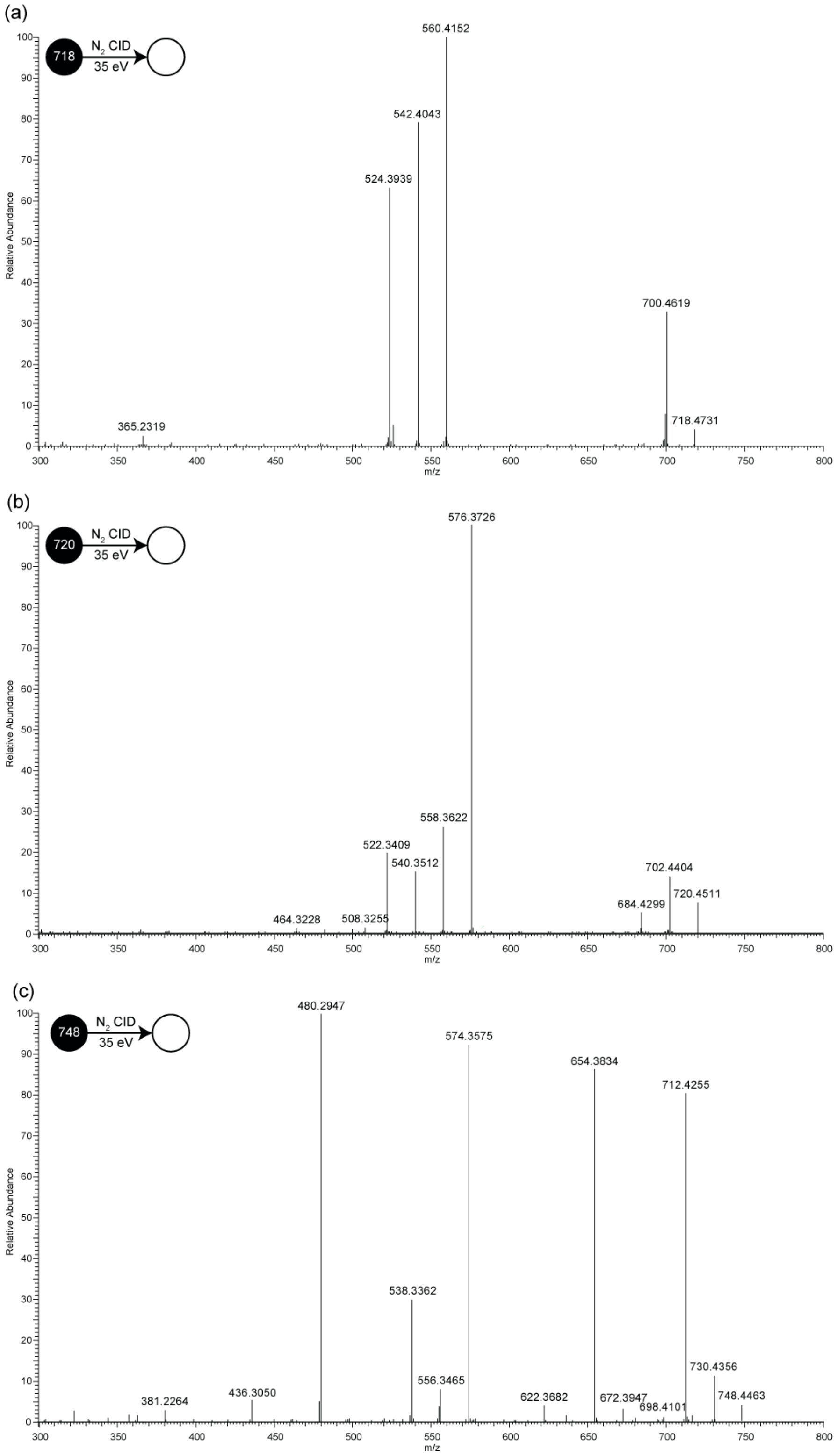

Figure S8. UHPLC-ESI-CID-MS/MS spectra of Erythromycin co-metabolites. Spectrum (a) is of Ery718, spectrum (b) is of Ery720 and spectrum (c) is of Ery748. The collision energies are displayed on the spectra.

Table S3. Table of observed *m/z*, formulae, theoretical *m/z*, error (ppm) and ion identification from the MS^n analysis of protonated EryA.

**Observed *m/z***	**Formula**	**Theoretical *m/z***	**Error (ppm)**	**Identification**
Figure 2(a) - MS^2 734				
734.4679	$C_{37}H_{68}NO_{13}^+$	734.4685	0.82	PI
716.4575	$C_{37}H_{66}NO_{12}^+$	716.4580	0.70	734 loss of H_2O
698.4467	$C_{37}H_{64}NO_{11}^+$	698.4474	1.00	716 loss of H_2O
576.3738	$C_{29}H_{54}NO_{10}^+$	576.3742	0.69	734 loss of cladinose
558.3632	$C_{29}H_{52}NO_9^+$	558.3637	0.90	576 loss of H_2O
540.3525	$C_{29}H_{50}NO_8^+$	540.3531	1.11	558 loss of H_2O
522.3421	$C_{29}H_{48}NO_7^+$	522.3425	0.77	540 loss of H_2O
Figure 2(b) - MS^2 734 > MS^3 716				
716.4570	$C_{37}H_{66}NO_{12}^+$	716.4580	1.40	PI
698.4464	$C_{37}H_{64}NO_{11}^+$	698.4474	1.43	716 loss of H_2O
658.4151	$C_{34}H_{60}NO_{11}^+$	658.4161	1.52	716 loss of propionate (C_3H_6O)
558.3627	$C_{29}H_{52}NO_9^+$	558.3637	1.79	716 loss of cladinose
540.3522	$C_{29}H_{50}NO_8^+$	540.3531	1.67	558 loss of H_2O
522.3417	$C_{29}H_{48}NO_7^+$	522.3425	1.53	540 loss of H_2O
500.3210	$C_{26}H_{46}NO_8^+$	500.3218	1.60	558 loss of propionate (C_3H_6O)
482.3106	$C_{26}H_{44}NO_7^+$	482.3112	1.24	500 loss of H_2O
464.3001	$C_{26}H_{42}NO_6^+$	464.3007	1.29	482 loss of H_2O
408.2740	$C_{23}H_{28}NO_5^+$	408.2744	0.98	464 loss of C_3H_4O
365.2322	$C_{21}H_{33}O_5^+$	365.2323	0.27	522 loss of desosamine
Figure 2(c) - MS^2 734 > MS^3 698				
698.4460	$C_{37}H_{64}NO_{11}^+$	698.4474	2.00	PI
522.3415	$C_{29}H_{48}NO_7^+$	522.3425	1.91	698 loss of cladinose-OH
464.3003	$C_{26}H_{42}NO_6^+$	464.3007	0.86	522 loss of propionate (C_3H_6O)
408.2746	$C_{23}H_{28}NO_5^+$	408.2744	0.49	464 loss of C_3H_4O
365.2325	$C_{21}H_{33}O_5^+$	365.2323	0.55	522 loss of desosamine
347.2220	$C_{21}H_{32}O_4^+$	365.2217	1.92	365 loss of H_2O
342.2279	$C_{18}H_{32}NO_5^+$	342.2275	1.17	464 loss of $C_8H_{10}O$

Figure 3(a) - MS[2] 734 > MS[3] 522				
522.3413	$C_{29}H_{48}NO_7^+$	522.3425	2.30	PI
464.2998	$C_{26}H_{42}NO_6^+$	464.3007	1.94	522 loss of propionate (C_3H_6O)
408.2740	$C_{23}H_{28}NO_5^+$	408.2744	0.98	464 loss of C_3H_4O
342.2272	$C_{18}H_{32}NO_5^+$	342.2275	0.87	464 loss of $C_8H_{10}O$
233.1540	$C_{15}H_{21}O_2^+$	233.1536	1.72	408 loss of des-OH
158.1177	$C_8H_{16}NO_2^+$	158.1176	0.63	[desosamine+H]$^+$
Figure 3(b) - MS[2] 734 > MS[3] 522 > MS[4] 464				
464.2997	$C_{26}H_{42}NO_6^+$	464.3007	2.15	PI
420.3107	$C_{25}H_{42}NO_4^+$	420.3108	0.24	464 loss of ?
408.2739	$C_{23}H_{28}NO_5^+$	408.2744	1.22	464 loss of C_3H_4O
342.2271	$C_{18}H_{32}NO_5^+$	342.2275	1.17	464 loss of $C_8H_{10}O$
158.1176	$C_8H_{16}NO_2^+$	158.1176	0.00	[desosamine+H]$^+$
Figure 3(c) - MS[2] 734 > MS[3] 522 > MS[4] 408				
408.2739	$C_{23}H_{28}NO_5^+$	408.2744	1.22	PI
286.2012	$C_{15}H_{28}NO_4^+$	286.2013	0.35	408 loss of ?
272.1855	$C_{14}H_{26}NO_4^+$	272.1856	0.37	408 loss of ?
233.1534	$C_{15}H_{21}O_2^+$	233.1536	0.86	408 loss of des-OH
176.1281	$C_8H_{18}NO_3^+$	176.1281	0.00	[desosamine+H]$^+$
Figure S5(a) - MS[2] 734 > MS[3] 576				
576.3729	$C_{29}H_{54}NO_{10}^+$	576.3742	2.26	PI
558.3625	$C_{29}H_{52}NO_9^+$	558.3637	2.15	576 loss of H_2O
540.3519	$C_{29}H_{50}NO_8^+$	540.3531	2.22	558 loss of H_2O
522.3414	$C_{29}H_{48}NO_7^+$	522.3425	2.11	540 loss of H_2O
500.3209	$C_{26}H_{46}NO_8^+$	500.3218	1.80	558 loss of propionate (C_3H_6O)
482.3105	$C_{26}H_{44}NO_7^+$	482.3112	1.45	500 loss of H_2O
464.3001	$C_{26}H_{42}NO_6^+$	464.3007	1.29	482 loss of H_2O
444.2951	$C_{23}H_{42}NO_7^+$	444.2956	1.13	500 loss of C_3H_4O
408.2744	$C_{23}H_{28}NO_5^+$	408.2744	0.00	464 loss of C_3H_4O
383.2426	$C_{21}H_{35}O_6^+$	383.2428	0.52	576 loss of desosamine / H_2O
365.2320	$C_{21}H_{33}O_5^+$	365.2323	0.82	383 loss of H_2O
347.2220	$C_{21}H_{31}O_4^+$	347.2217	0.86	365 loss of H_2O
325.2008	$C_{18}H_{29}O_5^+$	325.2010	0.62	383 loss of propionate (C_3H_6O)
307.1908	$C_{18}H_{27}O_4^+$	307.1904	1.30	325 loss of H_2O

Figure S5(b) - MS^2 734 > MS^3 558				
558.3623	$C_{29}H_{52}NO_9^+$	558.3637	2.51	PI
540.3518	$C_{29}H_{50}NO_8^+$	540.3531	2.41	558 loss of H_2O
522.3412	$C_{29}H_{48}NO_7^+$	522.3425	2.49	540 loss of H_2O
500.3206	$C_{26}H_{46}NO_8^+$	500.3218	2.40	558 loss of propionate (C_3H_6O)
482.3106	$C_{26}H_{44}NO_7^+$	482.3112	1.24	500 loss of H_2O
Figure S5(c) - MS^2 734 > MS^3 540				
540.3519	$C_{29}H_{50}NO_8^+$	540.3531	2.22	558 loss of H_2O
522.3413	$C_{29}H_{48}NO_7^+$	522.3425	2.30	540 loss of H_2O
482.3106	$C_{26}H_{44}NO_7^+$	482.3112	1.24	540 loss of propionate (C_3H_6O)
158.1176	$C_8H_{16}NO_2^+$	158.1176	0.00	desosamine
Figure S6(a) - MS^2 734 > MS^3 558 > MS^4 500				
500.3212	$C_{26}H_{46}NO_8^+$	500.3218	1.20	PI
482.3112	$C_{26}H_{44}NO_7^+$	482.3112	0.00	500 loss of H_2O
158.1178	$C_8H_{16}NO_2^+$	158.1176	1.27	desosamine
Figure S6(b) - MS^2 734 > MS^3 522 > MS^4 342				
342.2271	$C_{18}H_{32}NO_5^+$	342.2275	1.17	PI
298.2374	$C_{17}H_{32}NO_3^+$	298.2377	1.01	342 loss of CO_2
158.1175	$C_8H_{16}NO_2^+$	158.1176	0.63	desosamine

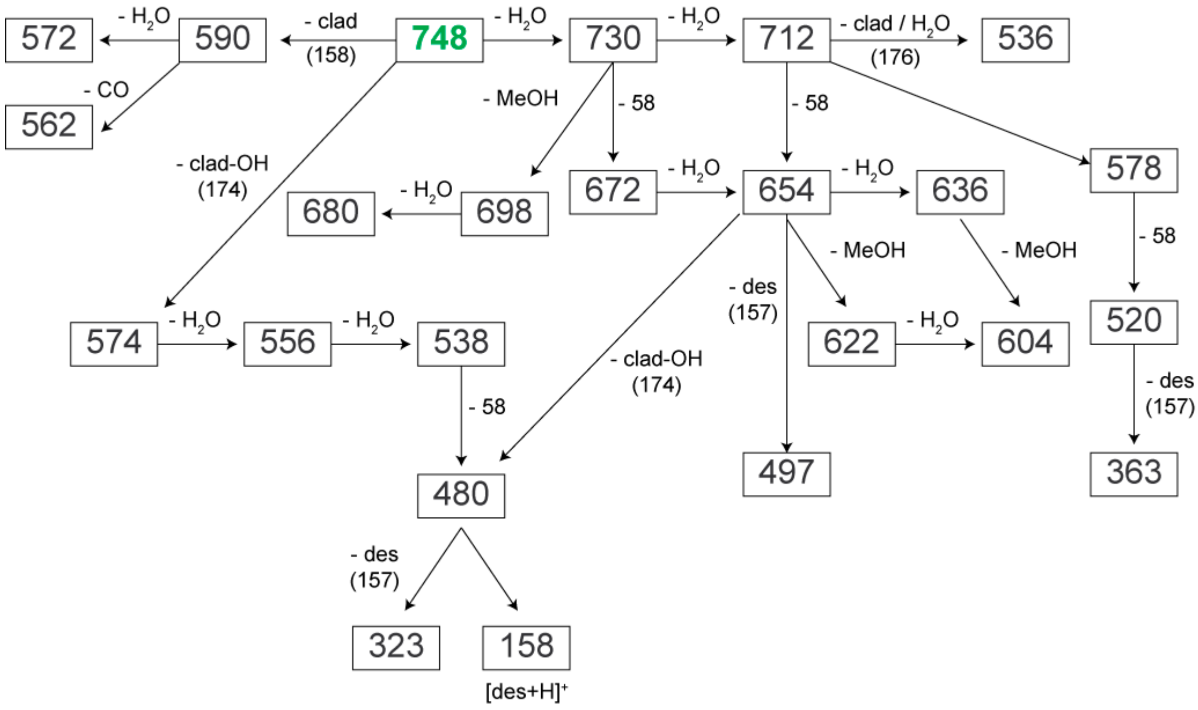

Figure S9. The fragmentation tree for the ESI-MS^n analysis of EryX ion (PI = *m/z* 748).

Table S4. Table of observed *m/z*, formulae, theoretical *m/z*, error (ppm) and ion identification from the MS^n analysis of protonated EryX.

**Observed *m/z***	**Formula**	**Theoretical *m/z***	**Error (ppm)**	**Identification**
Figure 4(a) - MS^2 748				
748.4468	$C_{37}H_{66}NO_{14}^+$	748.4478	1.34	PI
730.4362	$C_{37}H_{64}NO_{13}^+$	730.4372	1.37	748 loss of H_2O
712.4260	$C_{37}H_{62}NO_{12}^+$	712.4267	0.98	730 loss of H_2O
698.4101	$C_{36}H_{60}NO_{12}^+$	698.4110	1.29	730 loss of MeOH
680.3996	$C_{36}H_{58}NO_{11}^+$	680.4004	1.18	698 loss of H_2O
672.3942	$C_{34}H_{58}NO_{12}^+$	672.3954	1.78	720 loss of propionate (C_3H_6O)
654.3838	$C_{34}H_{56}NO_{11}^+$	654.3848	1.53	672 loss of H_2O
590.3889	$C_{30}H_{56}NO_{10}^+$	590.3899	1.69	748 loss of cladinose
574.3580	$C_{29}H_{52}NO_{10}^+$	574.3586	1.04	748 loss of cladinose-OH
572.3787	$C_{30}H_{54}NO_9^+$	572.3793	1.05	590 loss of H_2O
562.3942	$C_{29}H_{56}NO_9^+$	562.3950	1.42	590 loss of CO
556.3471	$C_{29}H_{50}NO_9^+$	556.3480	1.62	574 loss of H_2O
538.3366	$C_{29}H_{48}NO_8^+$	538.3374	1.49	556 loss of H_2O
480.2950	$C_{26}H_{42}NO_7^+$	480.2950	1.25	654 loss of cladinose-OH
436.3054	$C_{25}H_{42}NO_5^+$	436.3057	0.69	480 loss of CO_2
363.2164	$C_{21}H_{31}O_5^+$	363.2166	0.55	538 loss of desosamine / H_2O
Figure 4(b) - MS^2 748 > MS^3 712				
712.4260	$C_{37}H_{62}NO_{12}^+$	712.4257	0.42	PI
654.3838	$C_{34}H_{56}NO_{11}^+$	654.3835	0.46	712 loss of propionate (C_3H_6O)
578.3313	$C_{31}H_{48}NO_9^+$	578.3324	1.90	712 loss of $C_6H_{14}O_3$
538.3363	$C_{29}H_{48}NO_8^+$	538.3374	2.04	712 loss of cladinose
536.3572	$C_{30}H_{50}NO_7^+$	536.3582	1.86	712 loss of $C_7H_{12}O_5$
520.3254	$C_{28}H_{42}NO_8^+$	520.3269	2.88	578 loss of propionate (C_3H_6O)
480.2947	$C_{26}H_{42}NO_7^+$	480.2950	0.62	654 loss of cladinose-OH
436.3050	$C_{25}H_{42}NO_5^+$	436.3057	1.60	480 loss of CO_2
363.2162	$C_{21}H_{31}O_5^+$	363.2166	1.10	520 loss of desosamine

Figure 4(c) - MS^2 748 > MS^3 712 > MS^4 654				
654.3832	$C_{34}H_{56}NO_{11}^+$	654.3835	0.46	PI
636.3728	$C_{34}H_{54}NO_{10}^+$	636.3742	2.20	654 loss of H_2O
622.3571	$C_{33}H_{52}NO_{10}^+$	622.3586	2.41	654 loss of MeOH
578.3313	$C_{31}H_{48}NO_9^+$	578.3324	1.90	654 loss of $C_3H_8O_2$
497.3103	$C_{27}H_{45}O_8^+$	497.3109	1.21	654 loss of desosamine
480.2945	$C_{26}H_{42}NO_7^+$	480.2950	1.04	654 loss of cladinose-OH
462.2840	$C_{26}H_{40}NO_6^+$	462.2850	2.16	From *m/z* 654
358.2220	$C_{18}H_{32}NO_6^+$	358.2224	1.12	654 loss of $C_{16}H_{24}O_5$
340.2114	$C_{18}H_{30}NO_5^+$	340.2118	1.18	358 loss of H_2O
Figure S7 - MS^2 748 > MS^3 712 > MS^4 480				
480.2942	$C_{26}H_{42}NO_7^+$	480.2950	1.67	PI
323.1848	$C_{18}H_{27}O_5^+$	323.1853	1.55	480 loss of desosamine
158.1174	$C_8H_{16}NO_2^+$	158.1176	1.27	desosamine

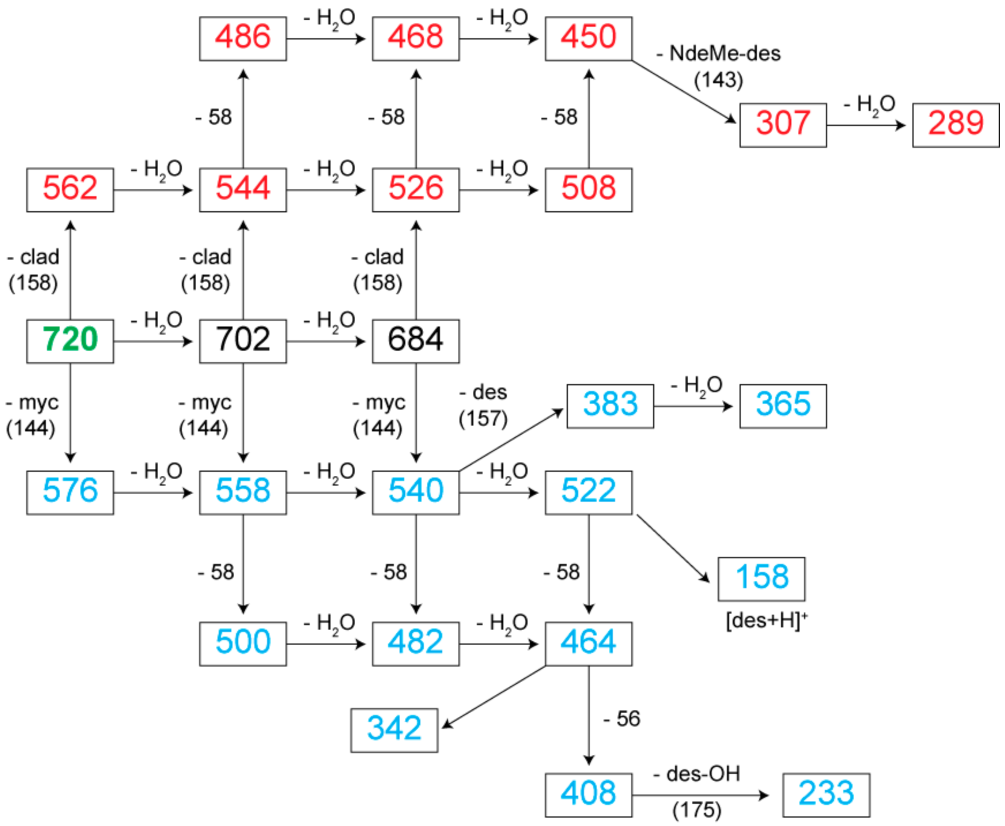

Figure S10. The fragmentation tree for the ESI-MS^n analysis of EryY ion (PI = *m/z* 720).

Table S5. Table of observed *m/z*, formulae, theoretical *m/z*, error (ppm) and ion identification from the MS^n analysis of protonated EryY. Red and blue are used to indicate the product ions from the two isomers.

**Observed *m/z***	**Formula**	**Theoretical *m/z***	**Error (ppm)**	**Identification**
Figure 7(a) - MS^2 720				
720.4512	$C_{36}H_{66}NO_{13}^+$	720.4529	2.36	PI
702.4407	$C_{36}H_{64}NO_{12}^+$	702.4423	2.28	720 loss of H_2O
684.4301	$C_{36}H_{62}NO_{11}^+$	684.4317	2.34	702 loss of H_2O
588.3729	$C_{30}H_{54}NO_{10}^+$	588.3742	2.21	720 loss of $C_6H_{12}O_3$
576.3729	$C_{29}H_{54}NO_{10}^+$	576.3742	2.26	720 loss of mycarose
562.3573	$C_{28}H_{52}NO_{10}^+$	562.3586	2.31	720 loss of cladinose
558.3625	$C_{29}H_{52}NO_9^+$	558.3637	2.15	576 loss of H_2O
544.3466	$C_{28}H_{50}NO_9^+$	544.3480	2.57	562 loss of H_2O
540.3518	$C_{29}H_{50}NO_8^+$	540.3531	2.41	558 loss of H_2O
526.3362	$C_{28}H_{48}NO_8^+$	526.3374	2.28	544 loss of H_2O
522.3412	$C_{29}H_{48}NO_7^+$	522.3425	2.49	540 loss of H_2O
508.3257	$C_{28}H_{46}NO_7^+$	508.3269	2.36	526 loss of H_2O
500.3207	$C_{26}H_{46}NO_8^+$	500.3218	2.20	558 loss of propionate (C_3H_6O)
486.3051	$C_{25}H_{44}NO_8^+$	486.3061	2.06	544 loss of propionate (C_3H_6O)
482.3103	$C_{26}H_{44}NO_7^+$	482.3112	1.87	540 loss of propionate (C_3H_6O)
468.2947	$C_{25}H_{42}NO_7^+$	468.2956	1.92	486 loss of H_2O
464.3000	$C_{26}H_{42}NO_6^+$	464.3007	1.51	522 loss of propionate (C_3H_6O)
450.2842	$C_{25}H_{40}NO_6^+$	450.2850	1.78	468 loss of H_2O
408.2738	$C_{23}H_{28}NO_5^+$	408.2744	1.47	464 loss of C_3H_4O
383.2422	$C_{21}H_{35}O_6^+$	383.2428	1.57	540 loss of desosamine
365.2317	$C_{21}H_{33}O_5^+$	365.2323	1.64	383 loss of H_2O
325.2006	$C_{18}H_{29}O_5^+$	325.2010	1.23	468 loss of NdeMe-desosamine
307.1901	$C_{18}H_{27}O_4^+$	307.1904	1.00	450 loss of NdeMe-desosamine
289.1797	$C_{18}H_{25}O_3^+$	289.1798	1.34	307 loss of H_2O

Figure 7(b) - MS^2 720 > MS^3 702				
702.4408	$C_{36}H_{64}NO_{12}^+$	702.4423	2.14	PI
684.4302	$C_{36}H_{62}NO_{11}^+$	684.4317	2.19	702 loss of H_2O
644.3987	$C_{33}H_{58}NO_{11}^+$	644.4004	2.64	702 loss of propionate (C_3H_6O)
558.3625	$C_{29}H_{52}NO_9^+$	558.3637	2.15	702 loss of mycarose
544.3467	$C_{28}H_{50}NO_9^+$	544.3480	2.39	702 loss of cladinose
540.3518	$C_{29}H_{50}NO_8^+$	540.3531	2.41	558 loss of H_2O
526.3362	$C_{28}H_{48}NO_8^+$	526.3374	2.28	544 loss of H_2O
522.3413	$C_{29}H_{48}NO_7^+$	522.3425	2.30	540 loss of H_2O
508.3256	$C_{28}H_{46}NO_7^+$	508.3269	2.56	526 loss of H_2O
365.2316	$C_{21}H_{33}O_5^+$	365.2323	1.92	522 loss of desosamine
Figure 7(c) - MS^2 720 > MS^3 576> MS^4 522				
522.3413	$C_{29}H_{48}NO_7^+$	522.3425	2.30	PI
464.2993	$C_{26}H_{42}NO_6^+$	464.3007	3.02	522 loss of propionate (C_3H_6O)
408.2736	$C_{23}H_{28}NO_5^+$	408.2744	1.96	464 loss of C_3H_4O
342.2268	$C_{18}H_{32}NO_5^+$	342.2275	2.05	464 loss of $C_8H_{10}O$
233.1533	$C_{15}H_{21}O_2^+$	233.1536	1.29	408 loss of des-OH
158.1175	$C_8H_{16}NO_2^+$	158.1176	0.63	[desosamine+H]$^+$

Table S6. Table of observed *m/z*, formulae, theoretical *m/z*, error (ppm) and ion identification from the MS^n analysis of protonated EryZ.

**Observed *m/z***	**Formula**	**Theoretical *m/z***	**Error (ppm)**	**Identification**
Figure 10(a) - MS^2 718				
718.4723	$C_{37}H_{68}NO_{12}^+$	718.4736	1.81	PI
700.4616	$C_{37}H_{66}NO_{11}^+$	700.4630	2.00	718 loss of H_2O
560.4142	$C_{30}H_{58}NO_8^+$	560.4157	2.68	718 loss of cladinose
542.4039	$C_{30}H_{56}NO_7^+$	542.4051	2.21	560 loss of H_2O
524.3941	$C_{30}H_{54}NO_6^+$	524.3946	0.95	542 loss of H_2O
365.2318	$C_{21}H_{33}O_5^+$	365.2323	1.37	720 loss of propionate (C_3H_6O)
Figure 10(b) - MS^2 718 > MS^3 542				
542.4040	$C_{30}H_{56}NO_7^+$	542.4051	2.03	PI
524.3940	$C_{30}H_{54}NO_6^+$	524.3946	1.14	542 loss of H_2O
367.2841	$C_{22}H_{39}O_4^+$	367.2843	0.54	524 loss of desosamine
349.2732	$C_{22}H_{37}O_3^+$	349.2737	1.43	367 loss of H_2O
158.1176	$C_8H_{16}NO_2^+$	158.1176	0.00	[desosamine+H]$^+$

Table S7. Table of observed *m/z*, formulae, theoretical *m/z*, error (ppm) and ion identification from the UHPLC-MS/MS analysis of protonated Ery716 isomers.

**Observed *m/z***	**Formula**	**Theoretical *m/z***	**Error (ppm)**	**Identification**
Figure 13(a) - MS^2 716A				
716.4575	$C_{37}H_{66}NO_{12}^+$	716.4580	0.70	PI
698.4472	$C_{37}H_{64}NO_{11}^+$	698.4474	0.29	716 loss of H_2O
658.4157	$C_{34}H_{60}NO_{11}^+$	658.4161	0.61	716 loss of propionate (C_3H_6O)
640.4052	$C_{34}H_{58}NO_{10}^+$	640.4055	0.47	658 loss of H_2O
558.3633	$C_{29}H_{52}NO_9^+$	558.3637	0.72	716 loss of cladinose
540.3527	$C_{29}H_{50}NO_8^+$	540.3531	0.74	558 loss of H_2O
522.3420	$C_{29}H_{48}NO_7^+$	522.3425	0.96	540 loss of H_2O
500.3215	$C_{26}H_{46}NO_8^+$	500.3218	0.60	558 loss of propionate (C_3H_6O)
482.3109	$C_{26}H_{44}NO_7^+$	482.3112	0.62	500 loss of H_2O
464.3002	$C_{26}H_{42}NO_6^+$	464.3007	1.08	482 loss of H_2O
408.2740	$C_{23}H_{28}NO_5^+$	408.2744	0.98	464 loss of C_3H_4O
342.2271	$C_{18}H_{32}NO_5^+$	342.2275	1.17	464 loss of $C_8H_{10}O$
Figure 13(b) - MS^2 716B				
716.4570	$C_{37}H_{66}NO_{12}^+$	716.4580	1.40	PI
698.4469	$C_{37}H_{64}NO_{11}^+$	698.4474	0.72	716 loss of H_2O
658.4155	$C_{34}H_{60}NO_{11}^+$	658.4161	0.91	716 loss of propionate (C_3H_6O)
558.3630	$C_{29}H_{52}NO_9^+$	558.3637	1.25	716 loss of cladinose
540.3526	$C_{29}H_{50}NO_8^+$	540.3531	0.93	558 loss of H_2O
500.3212	$C_{26}H_{46}NO_8^+$	500.3218	1.20	558 loss of propionate (C_3H_6O)
482.3107	$C_{26}H_{44}NO_7^+$	482.3112	1.04	500 loss of H_2O
365.2221	$C_{21}H_{33}O_5^+$	365.2323	0.55	540 loss of desosamine + H_2O
Figure 13(c) - MS^2 716C				
716.4572	$C_{37}H_{66}NO_{12}^+$	716.4580	1.12	PI
698.4470	$C_{37}H_{64}NO_{11}^+$	698.4474	0.57	716 loss of H_2O
558.3631	$C_{29}H_{52}NO_9^+$	558.3637	1.07	716 loss of cladinose
540.3525	$C_{29}H_{50}NO_8^+$	540.3531	1.11	558 loss of H_2O

Table S8. Table of observed *m/z*, formulae, theoretical *m/z*, error (ppm) and ion identification from the UHPLC-MS/MS analysis of protonated Ery750.

**Observed *m/z***	**Formula**	**Theoretical *m/z***	**Error (ppm)**	**Identification**
Figure 14 - MS^2 750				
750.4635	$C_{37}H_{68}NO_{14}^+$	750.4634	0.13	PI
732.4531	$C_{37}H_{66}NO_{13}^+$	732.4529	0.27	750 loss of H_2O
714.4420	$C_{37}H_{64}NO_{12}^+$	714.4423	0.42	732 loss of H_2O
592.4051	$C_{30}H_{58}NO_{10}^+$	592.4055	0.68	750 loss of cladinose
574.3944	$C_{30}H_{56}NO_9^+$	574.3950	1.04	592 loss of H_2O
556.3838	$C_{30}H_{54}NO_8^+$	556.3844	1.08	574 loss of H_2O
538.3732	$C_{30}H_{52}NO_7^+$	538.3738	1.11	556 loss of H_2O
516.3526	$C_{27}H_{50}NO_8^+$	516.3531	0.97	574 loss of propionate (C_3H_6O)
498.3419	$C_{27}H_{48}NO_7^+$	498.3425	1.20	516 loss of H_2O
480.3312	$C_{27}H_{46}NO_6^+$	480.3320	1.67	498 loss of H_2O
381.2632	$C_{22}H_{37}O_5^+$	381.2636	1.05	538 loss of desosamine

Table S9. Table of observed *m/z*, formulae, theoretical *m/z*, error (ppm) and ion identification from the UHPLC-MS/MS analysis of protonated Ery718, Ery720 and Ery748.

**Observed *m/z***	**Formula**	**Theoretical *m/z***	**Error (ppm)**	**Identification**
Figure S8(a) - MS^2 718				
718.4731	$C_{37}H_{68}NO_{12}^+$	718.4736	0.70	PI
700.4619	$C_{37}H_{66}NO_{11}^+$	700.4630	1.57	718 loss of H_2O
560.4152	$C_{30}H_{58}NO_8^+$	560.4157	0.89	718 loss of cladinose
542.4043	$C_{30}H_{56}NO_7^+$	542.4051	1.47	560 loss of H_2O
524.3939	$C_{30}H_{54}NO_6^+$	524.3946	1.33	542 loss of H_2O
365.2319	$C_{21}H_{33}O_5^+$	365.2323	1.10	720 loss of propionate (C_3H_6O)
Figure S8(b) - MS^2 720				
720.4511	$C_{36}H_{66}NO_{13}^+$	720.4529	2.50	PI
702.4404	$C_{36}H_{64}NO_{12}^+$	702.4423	2.71	720 loss of H_2O
684.4299	$C_{36}H_{62}NO_{11}^+$	684.4317	2.63	702 loss of H_2O
576.3726	$C_{29}H_{54}NO_{10}^+$	576.3742	2.78	720 loss of mycarose
558.3622	$C_{29}H_{52}NO_9^+$	558.3637	2.69	576 loss of H_2O
540.3512	$C_{29}H_{50}NO_8^+$	540.3531	3.52	558 loss of H_2O
522.3409	$C_{29}H_{48}NO_7^+$	522.3425	3.06	540 loss of H_2O

Figure S8(c) - MS[2] 748				
748.4463	$C_{37}H_{66}NO_{14}^+$	748.4478	2.00	PI
730.4356	$C_{37}H_{64}NO_{13}^+$	730.4372	2.19	748 loss of H_2O
712.4255	$C_{37}H_{62}NO_{12}^+$	712.4267	1.68	730 loss of H_2O
698.4101	$C_{36}H_{60}NO_{12}^+$	698.4110	1.29	730 loss of MeOH
672.3947	$C_{34}H_{58}NO_{12}^+$	672.3954	1.04	720 loss of propionate (C_3H_6O)
654.3834	$C_{34}H_{56}NO_{11}^+$	654.3848	2.14	672 loss of H_2O
574.3575	$C_{29}H_{52}NO_{10}^+$	574.3586	2.26	748 loss of cladinose-OH
556.3465	$C_{29}H_{50}NO_9^+$	558.3480	2.69	574 loss of H_2O
538.3362	$C_{29}H_{48}NO_8^+$	538.3374	2.23	556 loss of H_2O
480.2947	$C_{26}H_{42}NO_7^+$	480.2950	0.62	654 loss of cladinose-OH
436.3050	$C_{25}H4_{2}NO_5^+$	436.3057	1.60	480 loss of CO_2
381.2264	$C_{21}H_{33}O_6^+$	381.2272	2.10	538 loss of desosamine

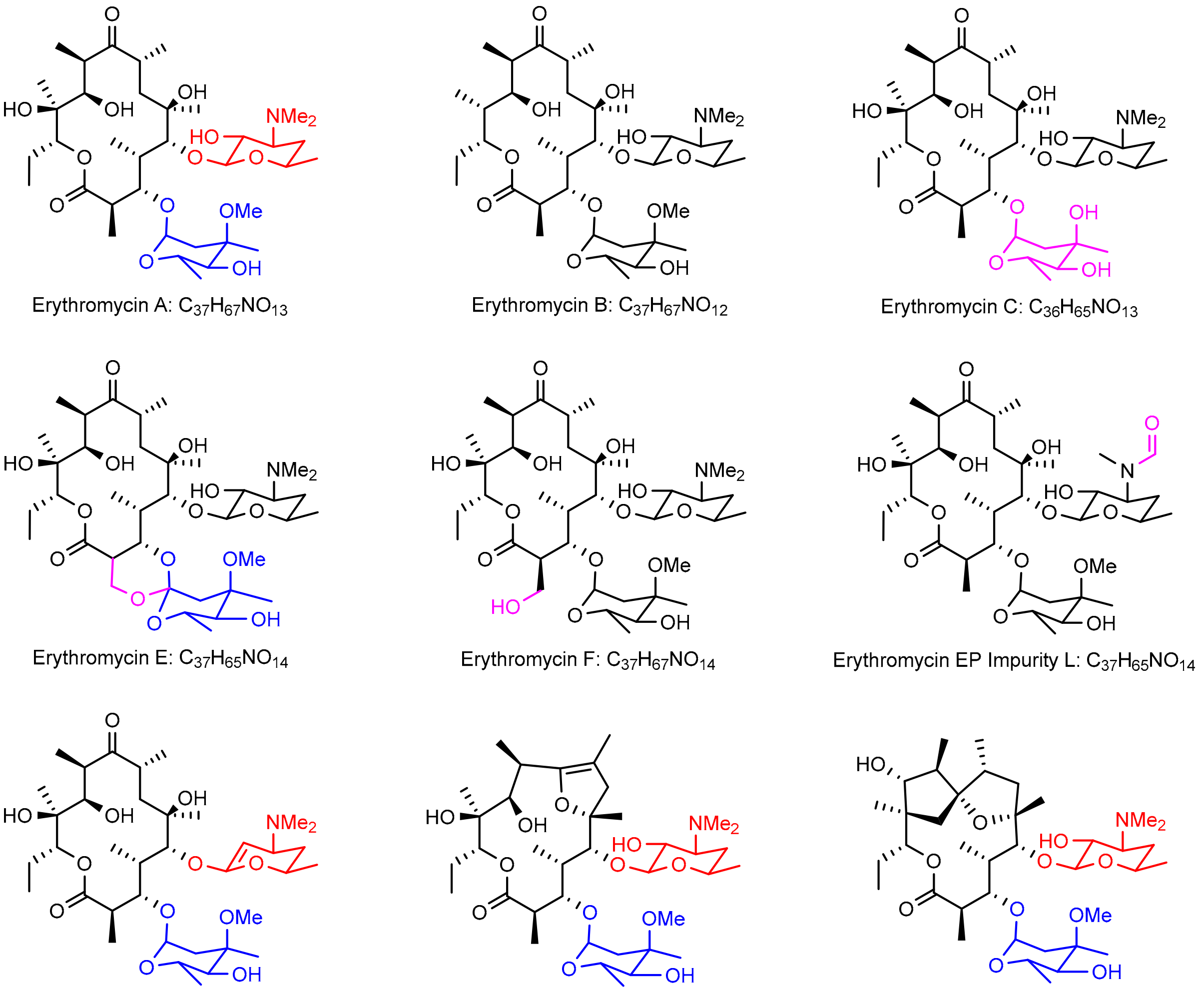

Figure S11. The proposed structures of all the erythromycin co-metabolites and degradation products detected in this study.

References:

S1. I. Kanfer, M.F. Skinner, R.B. Walker, *Analysis of macrolide antibiotics*. J. Chromatogr. A, 1998, 812, 255-286. DOI:10.1016/s0021-9673(98)00276-3.

S2. I. O. Kibwage, J. Hoogmartens, E. Roets, H. Vanderhaeghe, L. Verbist, M. Dubost, C. Pascal, P. Petitjean, G. Levol, *Antibacterial activities of erythromycins A, B, C, and D and some of their derivatives*. Antimicrob. Agents Chemother., 1985, 28, 630-633. DOI:10.1128/aac.28.5.630.

S3. H.A. Kirst, *Semi-synthetic derivatives of erythromycin. Prog. Med. Chem.,* 1993, 30, 57-88. DOI:10.1016/S0079-6468(08)70375-8.

S4. P.J. Atkins, T.O. Herbert, N.B. Jones, *Kinetic studies on the decomposition of erythromycin A in aqueous acidic and neutral buffers.* Int. J. Pharma., 1986, 30, 199-207. DOI:10.1016/0378-5173(86)90079-7.

S5. P. Kurath, P.H. Jones, R.S. Egan, T. J. Perun, *Acid degradation of erythromycin A and erythromycin B*. Experientia, 1971, 27, 362-362. DOI:10.1007/BF02137246.

S6. P.J. Gates, G.C. Kearney, R. Jones, P.F. Leadlay, J. Staunton, *Structural elucidation studies of erythromycins by electrospray tandem mass spectrometry*. R. Commun. Mass Spectrom., 1999, 13, 242-246. DOI:10.1002/(SICI)1097-0231(19990228)13:4<242::AID-RCM447>3.0.CO;2-B.

S7. G.C. Kearney, P.J. Gates, P.F. Leadlay, J. Staunton, R. Jones, *Structural elucidation studies of erythromycins by electrospray tandem mass spectrometry II.* R. Commun. Mass Spectrom., 1999, 13, 1650-1656. DOI:10.1002/(SICI)1097-0231(19990830)13:16<1650::AID-RCM693>3.0.CO;2-8.
